# Musculoskeletal pains and cardiovascular autonomic function in the general Northern Finnish population

**DOI:** 10.1186/s12891-019-2426-2

**Published:** 2019-01-31

**Authors:** Petteri Oura, Arto Hautala, Antti Kiviniemi, Juha Auvinen, Katri Puukka, Mikko Tulppo, Heikki Huikuri, Tapio Seppänen, Jaro Karppinen

**Affiliations:** 1 Faculty of Medicine, Center for Life Course Health Research, University of Oulu, P.O. Box 5000, FI-90014 Oulu, Finland; 2 Medical Research Center Oulu, Oulu University Hospital and University of Oulu, P.O. Box 5000, FI-90014 Oulu, Finland; 3 Faculty of Information Technology and Electrical Engineering, Center for Machine Vision and Signal Analysis, University of Oulu, P.O. Box 5000, FI-90014 Oulu, Finland; 4 Faculty of Medicine, Research Unit of Internal Medicine, University of Oulu, P.O. Box 5000, FI-90014 Oulu, Finland; 5 NordLab Oulu, Oulu University Hospital and Department of Clinical Chemistry, University of Oulu, P.O. Box 5000, FI-90014 Oulu, Finland; 6 Finnish Institute of Occupational Health, Aapistie 1, FI-90220 Oulu, Finland

**Keywords:** Autonomic nervous system, Cardiovascular autonomic function, Heart rate variability, Baroreflex sensitivity, Musculoskeletal pain, Multi-site pain

## Abstract

**Background:**

Heart rate variability (HRV) and baroreflex sensitivity (BRS) measurements provide means for the objective assessment of cardiovascular autonomic function. As previous studies have associated chronic pain with abnormal autonomic function, we aimed to characterize the relationship between the number of musculoskeletal pain sites (NPS), pain intensity, and cardiovascular autonomic function among the population-based Northern Finland Birth Cohort 1966.

**Methods:**

At the age of 46, cohort members self-reported their musculoskeletal pains (enabling the determination of NPS [0–8] and pain intensity [Numerical Rating Scale, NRS, 0–10]) and underwent clinical assessments of cardiovascular autonomic function in seated and standing positions (HRV variables: heart rate [HR] and root mean square of successive differences in beat-to-beat intervals [rMSSD] for the entire cohort; BRS variables: low-frequency systolic blood pressure variability [SBPV] and cross-spectral baroreflex sensitivity [BRS] for those attending the examination in Oulu, Finland). Extensive confounder data were also collected (body mass index, physical activity, smoking, Hopkins Symptom Checklist-25, comorbidities, and medications). The full samples included 4186 and 2031 individuals (HRV and BRS samples, respectively). Three subanalyses focused on individuals with intense and frequent pain, individuals with symptoms of depression and anxiety, and the relationship between pain intensity and autonomic parameters.

**Results:**

Linear regression models showed varying associations between NPS, pain intensity, and cardiovascular autonomic parameters. However, after all adjustments NPS was only associated with one outcome among women (BRS, standing: beta = − 0.015, *p* = 0.048) and two among men (HR, seated: beta = − 0.902, *p* = 0.003; HR, standing: beta = − 0.843, *p* = 0.014). Pain intensity was not associated with any outcome after full adjustments. Significant sex*pain interactions were found in the data.

**Conclusions:**

Our data suggest that musculoskeletal pain has, at most, a limited independent association with cardiovascular autonomic function. Future studies should carefully account for the potential confounders and sex interactions that this study revealed.

**Electronic supplementary material:**

The online version of this article (10.1186/s12891-019-2426-2) contains supplementary material, which is available to authorized users.

## Background

The sensation of pain, mediated by the peripheral and central nervous system to the cerebral cortex where it is ultimately perceived, serves as an indispensable protective function in the human body [1]. However, it notoriously commonly converts to disabling chronic conditions such as low back pain, fibromyalgia, or other complex pain syndromes [2, 3]. Although much remains to be studied in terms of the mechanisms of pain [1, 2], the interconnections between the autonomic nervous system (ANS) and pain have long been recognized [4]. Being able to adjust autonomic functions such as heart rate (HR), the ANS contributes to maintaining physiological equilibrium under changing conditions. Importantly, the ANS also regulates and modulates the perception of pain [5, 6].

Non-invasive methods for assessing cardiovascular autonomic function include measurements of vagally-mediated heart rate variability (HRV) and spontaneous baroreflex sensitivity (BRS), in which depressed vagal activity and augmented sympathetic activity suggest dysregulation [6–8]. Abnormal HRV and BRS parameters, indicating impaired cardiovascular autonomic function, seem to increase the risk of sudden cardiac death [7–11]. Together with various lifestyle-related factors and medical conditions [12–17], chronic pain has been associated with abnormal ANS function [6, 18], although there are also contradictory findings [19]. These considerations establish the need for further data on the relationship between pain characteristics and ANS function, not solely in patients with severe pain conditions but also from a wider perspective, including the general population with multiple pains. Potentially, this knowledge could be exploited to develop feasible tools for objectively quantifying the individual pain experience, to enhance health care resource allocation and ultimately, to improve pain treatment.

In this study we investigated the association between musculoskeletal pains and cardiovascular autonomic function in a large unselected sample of Northern Finns. At the age of 46, the participants self-reported the location, frequency and intensity of their pains with the aid of a questionnaire, and attended recordings of HRV and BRS, allowing the analysis of cardiovascular autonomic function. Based on previous findings regarding individuals suffering from chronic pain [6, 18], we hypothesized that an increasing number of pain sites (NPS) and increasing pain intensity would be associated with cardiovascular autonomic dysregulation in our general population sample.

## Methods

### Initiation and progression of the cohort study

**Fig. 1:**
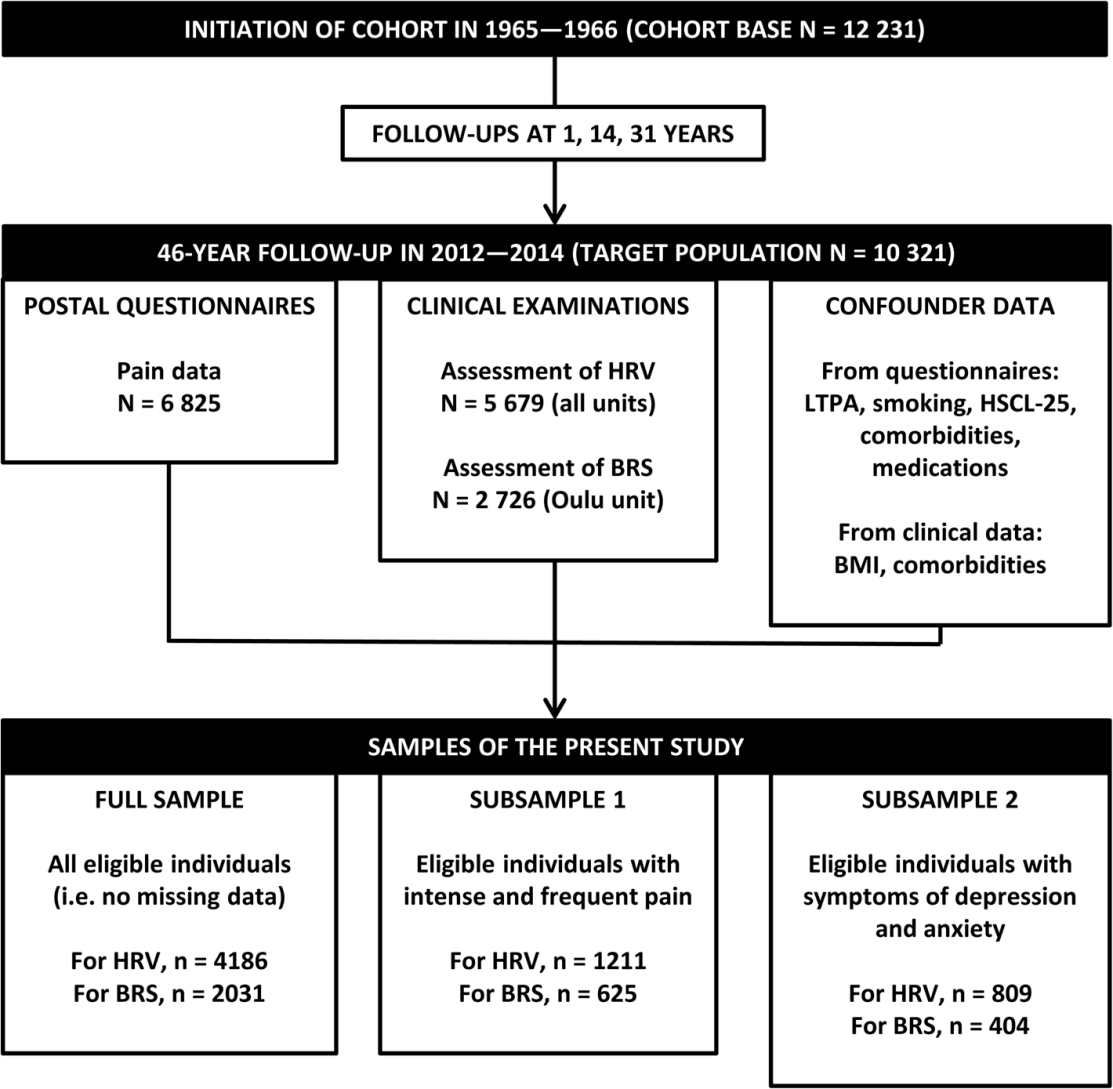
Flow chart of the study. *BMI* Body mass index, *BRS* Baroreflex sensitivity, *HRV* Heart rate variability, *HSCL-25* Hopkins Symptom Checklist-25, *LTPA* Leisure-time physical activity

We used a large middle-aged sample from the prospective, population-based Northern Finland Birth Cohort 1966 (NFBC1966) as our study population. The cohort was initiated in 1965–1966, when pregnant women who lived in the Northern Finnish provinces of Oulu and Lapland and had expected dates of delivery in the year 1966 (Jan 1 to Dec 31) were recruited into the cohort [20]. The cohort base initially covered up to 96% of births in the area (12,068 mothers and 12,231 children). The main data collections have been arranged when the cohort members (i.e., those born into the cohort) have been 1, 14, 31, and 46 years old. Data have been collected through postal questionnaires and clinical examinations. Figure 1 presents the progression of NFBC1966.

In 2012–2014, when the cohort members were 46 years old, postal questionnaires were sent to all those eligible (i.e., those alive with known contact details; *n* = 10,321), the response rate being 66% (*n* = 6825). Subsequently, those who were living in Finland were invited to participate in clinical examinations at laboratory units which were set up across the country by the NFBC project, with the attendance rate of 57% (*n* = 5861). The postal questionnaires included a pain survey, and the clinical examination included recordings of cardiovascular autonomic function, as specified below. Those who attended the clinical examinations in the Oulu laboratory unit were assessed for both HRV and BRS, whereas those attending the examination at laboratories outside Oulu were assessed for HRV only. From those who had attended the clinical examination at the 46-year follow-up, we excluded 1675 individuals due to missing data in the HRV analyses, and 3830 individuals due to missing data in the BRS analyses. The sample sizes and origins of the data are specified in Fig. 1.

### Assessment of musculoskeletal pains at age 46

Musculoskeletal pains were self-reported via a questionnaire that elicited the individual’s previous history of pain symptoms by the question: ‘Have you had any aches or pains in the following areas of your body within the last 12 months (yes/no)? 1) neck, 2) shoulders, 3) arms/elbows 4) wrists/hands 5) low back, 6) hips 7) knees, 8) ankles/feet’. The question was accompanied with an illustration defining these anatomical areas. Each positive response was followed by a subsequent question: ‘How often have you had aches or pains in this area during the last 12 months? 1) On 1–7 days, 2) On 8–30 days, 3) On more than 30 days but not daily, 4) Daily’. In addition, the participant was asked to estimate the overall intensity of their pains using a Numerical Rating Scale (NRS) from 0 (defined as ‘no pain’) to 10 (defined as ‘extremely severe pain’).

Based on the pain location data, we created a variable representing the number of anatomical sites at which the individual had experienced pain during the previous 12 months (NPS, 0–8). NPS is a widely used measure of musculoskeletal pain [21] that has been associated with, e.g., overall health and sleep quality in the general population [22]. In addition to the full study population, we were also interested in two subgroups: those with at least moderately intense and frequent pain (corresponding to an intensity of ≥5 on NRS [23, 24] and frequency of > 30 days/year), i.e., Subsample 1 and corresponding Subanalysis 1; and those with pain-related clinically relevant symptoms of depression and anxiety (corresponding to Hopkins Symptom Checklist-25 (HSCL-25) total mean score of ≥1.55 [25, 26]), i.e., Subsample 2 and corresponding Subanalysis 2 (Fig. 1). Subanalysis 3 evaluated the association between overall pain intensity (according to NRS) and autonomic function among the full study population.

### Assessment of cardiovascular autonomic function at age 46

We assessed cardiovascular autonomic function by means of HRV and BRS parameters at the 46-year clinical examinations. The detailed methodology behind the acquisition, inspection and processing of the cardiovascular ANS data is provided in earlier publications [15–17] which also introduced the HRV and BRS variables used here. For the assessment of HRV, we used an HR monitor (RS800CX, Polar Electro Oy, Kempele, Finland) to record R-R intervals (RRi) to an accuracy of 1 ms. For the assessment of BRS, we recorded standard lead-II electrocardiograms (ECG; Cardiolife, Nihon Kohden, Tokyo, Japan) together with ventilation rate (VR; nasal temperature probe MLT415/D by ADInstruments, Bella Vista, New South Wales, Australia) and blood pressure (BP; finger plethysmography by Nexfin, BMEYE Medical Systems, Amsterdam, the Netherlands), with a 1000 Hz sampling frequency (PowerLab 8/35, ADInstruments). At the measurement laboratory, we first ensured that the participant was correctly positioned (sitting position) and that the measurement devices were properly adjusted. We then allowed one minute for the participant’s HR to stabilize, after which we obtained a three-minute recording of data in a sitting position. Then, the participant stood up and remained standing for another three minutes while we recorded the data a second time. We analyzed the first 150 s of sitting data and the last 150 s of standing data.

For the analysis of HRV, we processed the RRi data by visual inspection (Hearts 1.2, University of Oulu, Oulu, Finland), replaced short artefacts and ectopic beats with the local average, and deleted longer sequences of noise or ectopy (≥ 10 consecutive defective beats). Recordings containing ≥80% of adequate data were accepted for further processing. Eligible HRV data in both sitting and standing positions were available for 5473 individuals (96% of those 5679 who attended the recording). From the eligible HRV data we calculated mean HR (bpm) and the root mean square of successive differences in RRi (rMSSD, ms), which represent cardiac vagal modulation [27].

For the analysis of BRS, we used custom-made software (Biosignal Processing Team, University of Oulu, Oulu, Finland) to process the ECG, BP and VR data. From the continuous ECG and BP recordings, we extracted systolic blood pressure (SBP) and RRi values, respectively. We replaced artefacts and ectopic beats using linear interpolation and then resampled at 2 Hz. Recordings with < 5% of defective data were accepted for further processing. We used the Savitzky-Golay method to delete very low frequency components (< 0.04 Hz) from the data, and we conducted fast Fourier transform (Welch method, segments of 128 samples with 50% overlap, length 1024 samples) to analyze the low frequency power of RRi and SBP oscillations (ms^2^, mmHg^2^) for the subsequent analysis of BRS by the alpha method, after ensuring a coherence of ≥0.5 between the low frequency oscillations in RRi and SBP [28]. Eligible BRS data in both sitting and standing positions were available for 2641 and 2617 individuals (97 and 96% of the 2726 who attended the recording), respectively. Low frequency SBP variability (SBPV, mmHg^2^), an estimate of peripheral sympathetic regulation [29], and cross-spectral BRS (ms/mmHg) are principal variables representing ANS function obtained by this protocol.

### Confounders

We assessed the participants’ body mass index (BMI) [17], leisure-time physical activity (LTPA) [15, 17], smoking [30], and symptoms of depression and anxiety [31] as potential confounders, together with comorbidities and medications influencing ANS function. BMI (kg/m^2^) was calculated on the basis of systematic height and weight measurements, which were obtained in the 46-year clinical examination. The frequency of LTPA was elicited in the 46-year questionnaires by asking: ‘How often do you participate in brisk physical activity (defined as causing at least some sweating and getting out of breath) during your leisure-time? 1) daily, 2) 4–6 times a week, 3) 2–3 times a week, 4) once a week, 5) 2–3 times a month, 6) once a month or less often’. We combined the first two and last two categories due to small group sizes. Lifetime smoking history was elicited in the 46-year questionnaire by the questions: ‘Have you ever smoked cigarettes (yes/no)?’ and ‘Do you currently smoke (yes/no)?’ As previously described [32], we created three categories according to the responses: non-smoker, former smoker and current smoker. Symptoms of depression and anxiety were assessed in the 46-year questionnaire using the acknowledged HSCL-25 tool [33, 34]. We used the total HSCL-25 mean score in the analysis. The participants were also asked to report all their medications, dietary supplements, and diseases diagnosed by a physician in the 46-year questionnaire. The routine use of antihypertensives and/or painkillers was regarded as confounding medication, while confounding comorbidities included diabetes mellitus, cardiovascular diseases and fibromyalgia. Data on diabetes mellitus were based not only on self-reports but also on a two-hour oral glucose tolerance test and the measurement of glycated hemoglobin in the 46-year follow-up, as described in our previous publication [16]. Age was not assessed as a confounder because the sample was coeval.

### Statistical analysis

We explored the characteristics of the sample by calculating the frequencies and percentages of the categorical variables, means and standard deviations (SD) of continuous variables with normal distributions, and the medians and interquartile ranges (IQR) of continuous variables with skewed distributions. NPS distribution was illustrated using histograms. We calculated the correlation between NPS and overall pain intensity using Spearman’s Rho (R).

We analyzed the association between musculoskeletal pain and cardiovascular autonomic parameters using linear regression models. The autonomic parameters served as independent outcomes, for which all models were run. The parameters which had skewed distributions were natural logarithm-transformed to Gaussian, as specified in Additional file 1. NPS was the primary predictor in all models except for Subanalysis 3 in which pain intensity (NRS) was used instead of NPS. Four sex-stratified models were run for each outcome: Model I (crude); Model II (adjusted for lifestyle); Model III (adjusted for comorbidities); and Model IV (adjusted for lifestyle and comorbidities). The variables used in the models are specified in the Results section; we have also summarized the outcomes, explanatory variables, variable coding, and regression models in Additional files 1, 2 and 3. Beta coefficients (β) of the predictors were collected with their 95% confidence intervals (CI). In addition to the Primary Analysis, we ran three subanalyses; the full sample and subsamples have been specified in the earlier part of the Methods section and in Fig. 1, and the subanalyses are further introduced in the Results section.

In addition to continuous modeling, the NPS data were also assessed using various categorizations (0,1,2,3,4,5,6,7,8; 0,1,2−3,4−5,6–8; 0,1−2,3−5,6–8) in order to rule out potential non-linear relationship patterns in the data and thus justify the linear modeling of NPS. The need for sex stratification was explored by running all models with pooled sexes and including the sex*NPS (or sex*NRS) interaction term in the models. The need for sex stratification was evaluated on the basis of the statistical significance of the interaction terms, taking into account the large sample size which would allow stratification without critically reducing the statistical power of the analyses.

The data were analyzed using SPSS version 25 (IBM, Armonk, NY, USA). *P* values of ≤ 0.05 were considered statistically significant.

### Ethical considerations

The study protocol followed the Declaration of Helsinki and was approved by the Ethical Committee of the Northern Ostrobothnia Hospital District in accordance with the Declaration of the World Medical Association. Each participant granted their written informed consent.

## Results

**Fig. 2:**
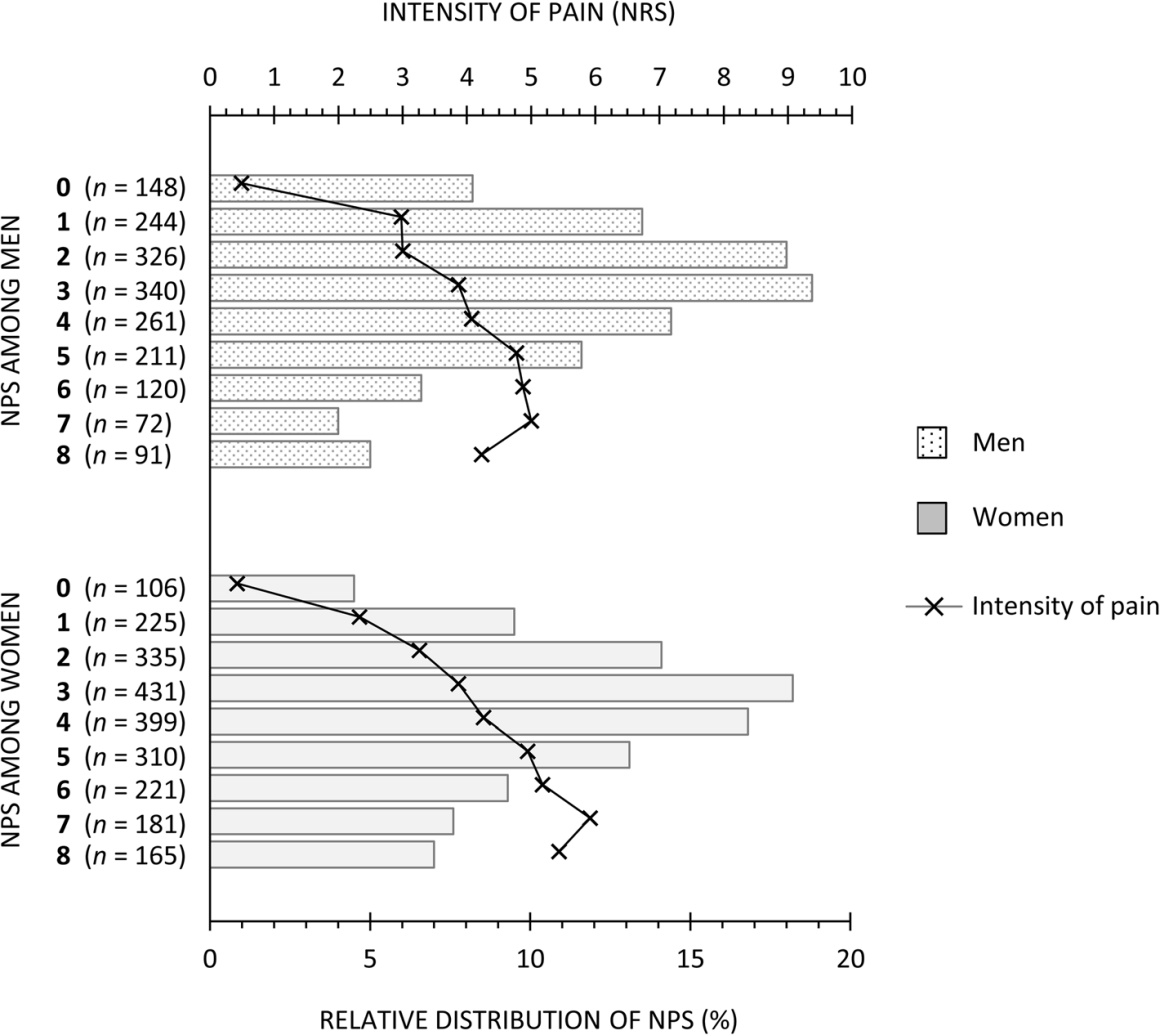
Distribution of NPS among men and women, and mean intensity of pain in NPS categories. *NPS* Number of pain sites, *NRS* Numerical Rating Scale (0–10)

The study sample consisted of 1813 men and 2373 women, whose detailed characteristics are presented in Table 1. The median NPS was 3 (interquartile range 2–5) among men and 4 (2–5) among women. Figure 2 presents the distribution of NPS among the sample, together with the mean reported intensity of pain in each NPS category. The overall intensity of pain (NRS) correlated positively with NPS (R = 0.382 among men and 0.430 among women, *p* < 0.001).

**Table 1:** Characteristics of the full sample, i.e., those with pain data, covariate data, and either HRV or BRS data (*n* = 4186)

Characteristic	Men	Women
Sex distribution^a^	43.3 (1813)	56.7 (2373)
BMI^b^ (kg/m^2^)	27.2 (4.1)	26.3 (5.0)
LTPA frequency (times/week)		
< 1^a^	29.8 (540)	23.2 (550)
1^a^	21.0 (381)	22.7 (538)
2–3^a^	34.4 (624)	37.5 (891)
≥ 4–6^a^	14.8 (268)	16.6 (394)
Smoking status		
Non-smoker^a^	46.9 (850)	57.6 (1367)
Former^a^	31.8 (576)	25.3 (601)
Current^a^	21.3 (387)	17.1 (405)
HSCL-25^c^ (total mean score)	1.20 (1.08–1.44)	1.24 (1.12–1.48)
Comorbidity associated with ANS function		
No^a^	90.3 (1638)	91.3 (2167)
Yes^a^	9.7 (175)	8.7 (206)
Medication associated with ANS function		
No^a^	84.5 (1532)	85.4 (2026)
Yes^a^	15.5 (281)	14.6 (347)
Characteristics of musculoskeletal pain		
NPS^c^	3 (2–5)	4 (2–5)
If pain, its intensity^c^ (NRS)	3 (2–6)	4 (2–6)
If pain, its frequency (days/year)		
0^a^	5.4 (98)	3.5 (82)
1–7^a^	15.4 (279)	10.7 (255)
8–30^a^	23.8 (432)	23.2 (551)
> 30 but not daily^a^	32.2 (583)	38.1 (904)
Daily^a^	20.5 (371)	23.5 (557)
Cardiovascular autonomic function		
HRV data (*n* = 1813 men, *n* = 2373 women)		
HR, seated^c^ (bpm)	70.2 (62.8–78.9)	71.3 (65.0–78.3)
HR, standing^c^ (bpm)	80.6 (72.2–89.4)	82.3 (74.6–90.6)
rMSSD, seated^c^ (ms)	19.9 (13.0–29.8)	23.8 (15.7–35.1)
rMSSD, standing^c^ (ms)	12.6 (8.2–18.5)	12.8 (8.4–19.2)
BRS data (*n* = 919 men, *n* = 1112 women)		
SBPV, seated^c^ (mmHg^2^)	5.70 (3.37–9.48)	5.46 (3.00–9.04)
SBPV, standing^c^ (mmHg^2^)	9.00 (5.07–15.83)	7.55 (4.21–12.65)
BRS, seated^c^ (ms/mmHg)	6.57 (4.76–9.35)	6.22 (4.41–8.49)
BRS, standing^c^ (ms/mmHg)	4.75 (3.22–6.67)	4.16 (3.01–5.90)

^a^Percent (number of individuals), ^b^Mean (standard deviation), ^c^Median (interquartile range)

*ANS* Autonomic nervous system, *BMI* Body mass index, *BRS* Baroreflex sensitivity, *HR* Heart rate, *HRV* Heart rate variability, *HSCL-25* Hopkins Symptom Checklist-25, *LTPA* Leisure-time physical activity, *NPS* Number of pain sites, *NRS* Numerical rating scale, *rMSSD* root mean square of the successive differences in R-R intervals, *SBPV* Systolic blood pressure variability

Non-linear relationships between NPS and cardiovascular autonomic parameters were not detected. However, the pooled-sex analyses showed statistically significant sex*NPS and sex*NRS interactions (*p* < 0.05, data not shown), and all models were consequently stratified by sex. The regression coefficients for the associations between NPS and cardiovascular autonomic parameters (i.e., HR, rMSSD, SBPV, BRS) are presented in Table 2 (Primary Analysis), Table 3 (Subanalysis 1), and Table 4 (Subanalysis 2). Correspondingly, the regression coefficients for the associations between pain intensity (NRS) and autonomic parameters are shown in Table 5 (Subanalysis 3). For clarity, we only provide the regression coefficients of the primary predictors (i.e., NPS/NRS) in the main manuscript and present the full models in Additional files 4, 5, 6, 7, 8, 9, 10 and 11.

**Table 2:** Primary Analysis. Results from sex-stratified linear regression models regarding the association between musculoskeletal pain sites (NPS) and cardiovascular autonomic function (HR, rMSSD, SBPV, BRS) in the full sample (for HR and rMSSD, *n* = 1813 men and 2373 women; for SBPV and BRS, *n* = 919 men and 1112 women). The complete models are presented in Additional files 4 and 5

	Model I	Model II	Model III	Model IV
	β for NPS [95% CI]	β for NPS [95% CI]	β for NPS [95% CI]	β for NPS [95% CI]
Men				
Outcome: HR, seated	0.125 [−0.140; 0.391]	− 0.117 [− 0.374; 0.139]	−0.016 [− 0.288; 0.256]	−0.184 [− 0.447; 0.079]
Outcome: HR, standing	0.188 [− 0.105; 0.481]	− 0.015 [− 0.304; 0.274]	0.036 [− 0.265; 0.337]	− 0.099 [− 0.395; 0.197]
Outcome: rMSSD, seated	− 0.010 [− 0.024; 0.004]	0.002 [− 0.011; 0.016]	−0.005 [− 0.019; 0.010]	0.004 [− 0.010; 0.018]
Outcome: rMSSD, standing	− 0.011 [− 0.025; 0.003]	0.000 [− 0.014; 0.014]	− 0.005 [− 0.019; 0.009]	0.002 [− 0.012; 0.016]
Outcome: SBPV, seated	− 0.001 [− 0.026; 0.024]	0.000 [− 0.025; 0.026]	0.006 [− 0.020; 0.032]	0.005 [− 0.021; 0.032]
Outcome: SBPV, standing	− 0.014 [− 0.039; 0.011]	− 0.012 [− 0.038; 0.014]	0.001 [− 0.025; 0.027]	− 0.001 [− 0.027; 0.026]
Outcome: BRS, seated	− 0.002 [− 0.019; 0.015]	0.011 [− 0.005; 0.027]	0.003 [− 0.015; 0.020]	0.013 [− 0.004; 0.029]
Outcome: BRS, standing	− 0.001 [− 0.019; 0.016]	0.011 [− 0.006; 0.028]	0.003 [− 0.015; 0.021]	0.013 [− 0.005; 0.030]
Women				
Outcome: HR, seated	**0.318 [0.119; 0.516]**	0.179 [− 0.017; 0.375]	**0.230 [0.024; 0.437]**	0.147 [− 0.055; 0.350]
Outcome: HR, standing	**0.299 [0.066; 0.531]**	0.203 [−0.029; 0.435]	**0.256 [0.014; 0.498]**	0.196 [−0.044; 0.436]
Outcome: rMSSD, seated	**−0.020 [− 0.032; − 0.009]**	**−0.011 [− 0.022; − 0.000]**	−0.011 [− 0.023; 0.001]	−0.006 [− 0.017; 0.006]
Outcome: rMSSD, standing	**− 0.016 [− 0.027; − 0.004]**	−0.009 [− 0.020; 0.003]	−0.008 [− 0.020; 0.004]	−0.004 [− 0.016; 0.008]
Outcome: SBPV, seated	− 0.001 [− 0.024; 0.022]	0.000 [− 0.023; 0.023]	0.003 [− 0.021; 0.027]	0.003 [− 0.021; 0.027]
Outcome: SBPV, standing	0.009 [− 0.014; 0.032]	0.011 [− 0.013; 0.034]	0.015 [− 0.010; 0.039]	0.014 [− 0.010; 0.038]
Outcome: BRS, seated	**−0.018 [− 0.032; − 0.004]**	−0.011 [− 0.025; 0.002]	−0.010 [− 0.024; 0.005]	−0.007 [− 0.021; 0.007]
Outcome: BRS, standing	**− 0.027 [− 0.042; − 0.012]**	**−0.019 [− 0.033; − 0.004]**	**−0.019 [− 0.034; − 0.003]**	**−0.015 [− 0.030; 0.000]**

Bold denotes statistical significance (*p* ≤ 0.05)

Model I (crude): NPS

Model II (adjusted for lifestyle): Model I + BMI + LTPA + smoking

Model III (adjusted for comorbidities): Model I + HSCL-25 + comorbidity + medication

Model IV (adjusted for lifestyle and comorbidities): Model II + Model III

*BMI* Body mass index, *BRS* Baroreflex sensitivity, *CI* Confidence interval, *HR* Heart rate, *HSCL-25* Hopkins Symptom Checklist-25, *LTPA* Leisure-time physical activity, *NPS* Number of pain sites, *P P* value for β, *rMSSD* Root mean square of successive differences in R-R intervals, *SBPV* Systolic blood pressure variability, *β* Beta coefficient

**Table 3:** Subanalysis 1. Results from sex-stratified linear regression models regarding the association between musculoskeletal pain sites (NPS) and cardiovascular autonomic function (HR, rMSSD, SBPV, BRS) in Subsample 1, i.e., individuals with intense (NRS ≥ 5) and frequent (> 30 days/year) pain (for HR and rMSSD, *n* = 450 men and 761 women; for SBPV and BRS, *n* = 250 men and 375 women). The complete models are presented in Additional files 6 and 7

	Model I	Model II	Model III	Model IV
	β for NPS [95% CI]	β for NPS [95% CI]	β for NPS [95% CI]	β for NPS [95% CI]
Men				
Outcome: HR, seated	− 0.448 [−1.046; 0.149]	**− 0.782 [− 1.371; − 0.193]**	**−0.663 [− 1.271; − 0.056]**	**−0.902 [− 1.501; − 0.302]**
Outcome: HR, standing	−0.349 [− 1.017; 0.319]	−0.660 [− 1.324; 0.003]	−0.643 [− 1.320; 0.034]	**−0.843 [− 1.515; − 0.171]**
Outcome: rMSSD, seated	0.005 [− 0.026; 0.037]	0.022 [− 0.009; 0.053]	0.011 [− 0.021; 0.044]	0.024 [− 0.008; 0.056]
Outcome: rMSSD, standing	0.005 [− 0.026; 0.037]	0.017 [− 0.014; 0.049]	0.013 [− 0.019; 0.045]	0.021 [− 0.011; 0.053]
Outcome: SBPV, seated	− 0.013 [− 0.066; 0.039]	− 0.010 [− 0.063; 0.043]	−0.009 [− 0.063; 0.046]	− 0.010 [− 0.064; 0.045]
Outcome: SBPV, standing	− 0.042 [− 0.094; 0.009]	− 0.030 [− 0.083; 0.002]	−0.029 [− 0.081; 0.024]	−0.022 [− 0.075; 0.030]
Outcome: BRS, seated	0.018 [− 0.017; 0.053]	**0.035 [0.001; 0.068]**	0.020 [− 0.016; 0.056]	0.034 [− 0.001; 0.068]
Outcome: BRS, standing	0.016 [− 0.021; 0.053]	0.030 [− 0.007; 0.066]	0.020 [− 0.018; 0.058]	0.029 [− 0.008; 0.067]
Women				
Outcome: HR, seated	**0.497 [0.108; 0.886]**	0.300 [−0.083; 0.683]	0.370 [− 0.027; 0.767]	0.239 [− 0.152; 0.629]
Outcome: HR, standing	**0.525 [0.058; 0.992]**	0.402 [−0.066; 0.869]	**0.482 [0.002; 0.963]**	0.396 [−0.083; 0.874]
Outcome: rMSSD, seated	**−0.032 [− 0.055; − 0.009]**	−0.019 [− 0.042; 0.004]	−0.018 [− 0.042; 0.005]	−0.011 [− 0.034; 0.012]
Outcome: rMSSD, standing	− 0.022 [− 0.046; 0.002]	−0.013 [− 0.037; 0.011]	−0.011 [− 0.035; 0.014]	−0.006 [− 0.031; 0.018]
Outcome: SBPV, seated	0.023 [− 0.020; 0.066]	0.030 [− 0.014; 0.073]	0.039 [− 0.006; 0.084]	0.041 [− 0.004; 0.086]
Outcome: SBPV, standing	0.029 [− 0.014; 0.072]	0.032 [− 0.011; 0.076]	0.040 [− 0.005; 0.086]	0.038 [− 0.007; 0.084]
Outcome: BRS, seated	**−0.033 [− 0.060; − 0.007]**	−0.024 [− 0.050; 0.002]	−0.023 [− 0.050; 0.004]	−0.019 [− 0.046; 0.008]
Outcome: BRS, standing	**− 0.038 [− 0.066; − 0.010]**	−0.026 [− 0.053; 0.002]	**−0.030 [− 0.058; − 0.001]**	−0.023 [− 0.050; 0.005]

Bold denotes statistical significance (*p* ≤ 0.05)

Model I (crude): NPS

Model II (adjusted for lifestyle): Model I + BMI + LTPA + smoking

Model III (adjusted for comorbidities): Model I + HSCL-25 + comorbidity + medication

Model IV (adjusted for lifestyle and comorbidities): Model II + Model III

*BMI* Body mass index, *BRS* Baroreflex sensitivity, *CI* Confidence interval, *HR* Heart rate, *HSCL-25* Hopkins Symptom Checklist-25, *LTPA* Leisure-time physical activity, *NPS* Number of pain sites, *NRS* Numerical rating scale, *P P* value for β, *rMSSD* Root mean square of successive differences in R-R intervals, *SBPV* Systolic blood pressure variability, *β* Beta coefficient

**Table 4:** Subanalysis 2. Results from sex-stratified linear regression models regarding the association between musculoskeletal pain sites (NPS) and cardiovascular autonomic function (HR, rMSSD, SBPV, BRS) in Subsample 2, i.e., individuals with clinically relevant symptoms of depression and anxiety (HSCL-25 score ≥ 1.55) (for HR and rMSSD, *n* = 302 men and 507 women; for SBPV and BRS, *n* = 163 men and 241 women). The complete models are presented in Additional files 8 and 9

	Model I	Model II	Model III	Model IV
	β for NPS [95% CI]	β for NPS [95% CI]	β for NPS [95% CI]	β for NPS [95% CI]
Men				
Outcome: HR, seated	0.040 [−0.669; 0.748]	− 0.209 [− 0.900; 0.482]	0.131 [− 0.579; 0.840]	−0.141 [− 0.840; 0.559]
Outcome: HR, standing	0.020 [− 0.768; 0.807]	− 0.180 [− 0.963; 0.603]	0.080 [− 0.714; 0.873]	− 0.140 [− 0.934; 0.653]
Outcome: rMSSD, seated	0.010 [− 0.027; 0.047]	0.026 [− 0.011; 0.062]	0.006 [− 0.031; 0.044]	0.024 [− 0.013; 0.061]
Outcome: rMSSD, standing	0.000 [− 0.036; 0.037]	0.012 [− 0.024; 0.049]	−0.002 [− 0.039; 0.035]	0.010 [− 0.028; 0.047]
Outcome: SBPV, seated	−0.011 [− 0.072; 0.049]	−0.008 [− 0.071; 0.054]	0.001 [− 0.061; 0.062]	0.004 [− 0.060; 0.068]
Outcome: SBPV, standing	0.009 [− 0.053; 0.070]	0.018 [− 0.045; 0.081]	0.015 [− 0.048; 0.079]	0.024 [− 0.041; 0.088]
Outcome: BRS, seated	0.023 [− 0.019; 0.065]	**0.042 [0.002; 0.082]**	0.012 [− 0.030; 0.054]	0.032 [− 0.009; 0.072]
Outcome: BRS, standing	0.027 [− 0.019; 0.072]	**0.047 [0.002; 0.092]**	0.016 [− 0.030; 0.062]	0.038 [− 0.008; 0.084]
Women				
Outcome: HR, seated	0.434 [− 0.018; 0.885]	0.265 [− 0.163; 0.694]	0.383 [− 0.072; 0.837]	0.267 [− 0.164; 0.698]
Outcome: HR, standing	**0.586 [0.059; 1.112]**	0.422 [− 0.086; 0.931]	**0.561 [0.030; 1.092]**	0.440 [− 0.071; 0.952]
Outcome: rMSSD, seated	−0.016 [− 0.042; 0.010]	−0.005 [− 0.030; 0.020]	−0.009 [− 0.036; 0.017]	−0.003 [− 0.028; 0.022]
Outcome: rMSSD, standing	− 0.018 [− 0.044; 0.009]	−0.007 [− 0.032; 0.018]	−0.010 [− 0.036; 0.016]	−0.004 [− 0.029; 0.021]
Outcome: SBPV, seated	0.022 [− 0.030; 0.073]	0.030 [− 0.022; 0.082]	0.022 [− 0.030; 0.075]	0.027 [− 0.026; 0.080]
Outcome: SBPV, standing	0.034 [− 0.015; 0.083]	0.039 [− 0.010; 0.088]	0.034 [− 0.016; 0.084]	0.037 [− 0.013; 0.088]
Outcome: BRS, seated	**−0.040 [− 0.070; − 0.010]**	**−0.030 [− 0.059; − 0.001]**	−0.029 [− 0.059; 0.001]	−0.024 [− 0.053; 0.005]
Outcome: BRS, standing	**− 0.053 [− 0.087; − 0.019]**	**−0.037 [− 0.068; − 0.006]**	**−0.038 [− 0.071; − 0.005]**	−0.028 [− 0.059; 0.003]

Bold denotes statistical significance (*p* ≤ 0.05)

Model I (crude): NPS

Model II (adjusted for lifestyle): Model I + BMI + LTPA + smoking

Model III (adjusted for comorbidities): Model I + comorbidity + medication

Model IV (adjusted for lifestyle and comorbidities): Model II + Model III

*BMI* Body mass index, *BRS* Baroreflex sensitivity, *CI* Confidence interval, *HR* Heart rate, *HSCL-25* Hopkins Symptom Checklist-25, *LTPA* Leisure-time physical activity, *NPS* Number of pain sites, *P P* value for β, *rMSSD* Root mean square of successive differences in R-R intervals, *SBPV* Systolic blood pressure variability, *β* Beta coefficient

**Table 5:** Subanalysis 3. Results from sex-stratified linear regression models regarding the association between between musculoskeletal pain intensity (according to NRS) and cardiovascular autonomic function (HR, rMSSD, SBPV, BRS) in the full sample (for HR and rMSSD, *n* = 1619 men and 2096 women; for SBPV and BRS, *n* = 832 men and 979 women). The complete models are presented in Additional files 10 and 11

	Model I	Model II	Model III	Model IV
	β for NRS [95% CI]	β for NRS [95% CI]	β for NRS [95% CI]	β for NRS [95% CI]
Men				
Outcome: HR, seated	**0.427 [0.200; 0.655]**	0.201 [−0.018; 0.421]	**0.348 [0.118; 0.578]**	0.172 [−0.050; 0.395]
Outcome: HR, standing	**0.460 [0.209; 0.710]**	**0.277 [0.030; 0.525]**	**0.375 [0.121; 0.629]**	0.237 [−0.014; 0.487]
Outcome: rMSSD, seated	**−0.024 [− 0.036; − 0.012]**	**−0.012 [− 0.023; − 0.000]**	**−0.020 [− 0.032; − 0.008]**	−0.011 [− 0.023; 0.001]
Outcome: rMSSD, standing	**− 0.020 [− 0.032; − 0.008]**	−0.011 [− 0.022; 0.001]	**−0.016 [− 0.028; − 0.004]**	−0.009 [− 0.021; 0.002]
Outcome: SBPV, seated	0.000 [− 0.020; 0.021]	0.002 [− 0.020; 0.023]	0.004 [− 0.017; 0.026]	0.004 [− 0.017; 0.026]
Outcome: SBPV, standing	−0.006 [− 0.027; 0.015]	−0.004 [− 0.025; 0.018]	0.004 [− 0.017; 0.026]	0.002 [− 0.019; 0.024]
Outcome: BRS, seated	**−0.019 [− 0.032; − 0.005]**	−0.006 [− 0.019; 0.007]	**−0.017 [− 0.031; − 0.003]**	−0.007 [− 0.020; 0.007]
Outcome: BRS, standing	− 0.014 [− 0.029; 0.000]	−0.003 [− 0.017; 0.011]	−0.013 [− 0.027; 0.002]	−0.003 [− 0.017; 0.012]
Women				
Outcome: HR, seated	0.128 [−0.037; 0.294]	0.048 [−0.115; 0.212]	0.046 [− 0.125; 0.218]	0.010 [− 0.159; 0.179]
Outcome: HR, standing	0.097 [− 0.097; 0.290]	0.055 [− 0.139; 0.248]	0.049 [− 0.153; 0.250]	0.035 [− 0.166; 0.236]
Outcome: rMSSD, seated	**−0.016 [− 0.026; − 0.006]**	**−0.010 [− 0.020; − 0.001]**	−0.009 [− 0.018; 0.001]	−0.006 [− 0.015; 0.004]
Outcome: rMSSD, standing	**− 0.014 [− 0.024; − 0.004]**	−0.010 [− 0.019; 0.001]	−0.008 [− 0.018; 0.002]	−0.006 [− 0.016; 0.004]
Outcome: SBPV, seated	− 0.013 [− 0.032; 0.006]	−0.011 [− 0.031; 0.008]	−0.010 [− 0.029; 0.010]	−0.009 [− 0.029; 0.010]
Outcome: SBPV, standing	− 0.002 [− 0.021; 0.017]	−0.001 [− 0.021; 0.018]	0.001 [− 0.019; 0.021]	0.001 [− 0.019; 0.021]
Outcome: BRS, seated	−0.006 [− 0.017; 0.006]	0.000 [− 0.011; 0.011]	0.001 [− 0.010; 0.013]	0.003 [− 0.008; 0.015]
Outcome: BRS, standing	**−0.016 [− 0.028; − 0.004]**	−0.009 [− 0.021; 0.003]	−0.009 [− 0.021; 0.004]	−0.006 [− 0.018; 0.006]

Bold denotes statistical significance (*p* ≤ 0.05)

Model I (crude): NRS

Model II (adjusted for lifestyle): Model I + BMI + LTPA + smoking

Model III (adjusted for comorbidities): Model I + HSCL-25 + comorbidity + medication

Model IV (adjusted for lifestyle and comorbidities): Model II + Model III

*BMI* Body mass index, *BRS* Baroreflex sensitivity, *CI* Confidence interval, *HR* Heart rate, *HSCL-25* Hopkins Symptom Checklist-25, *LTPA* Leisure-time physical activity, *NPS* Number of pain sites, *NRS* Pain intensity according to Numerical Rating Scale, *P* P value for β, *rMSSD* Root mean square of successive differences in R-R intervals, *SBPV* Systolic blood pressure variability, *β* Beta coefficient

Among women, NPS showed several statistically significant associations with cardiovascular autonomic parameters in the crude and partially adjusted models of the Primary Analysis, Subanalysis 1 and Subanalysis 2, but these associations were mostly attenuated by adjustments (Tables 2–4). Only in the Primary Analysis was one outcome (i.e., BRS, standing) consistently associated with NPS after full adjustments (β = − 0.015, *p* = 0.048). In Subanalysis 3, pain intensity showed several statistically significant crude associations with the autonomic parameters, but all of these became non-significant after full adjustments (Table 5).

Among men, some statistically significant associations between NPS and autonomic parameters were obtained from Subanalysis 1 and Subanalysis 2 (Tables 2–4), but only in Subanalysis 1 were two outcomes (i.e., HR, seated and standing) associated with NPS after full adjustments (HR, seated: β = − 0.902, *p* = 0.003; HR, standing: β = − 0.843, *p* = 0.014). In Subanalysis 3, pain intensity showed several statistically significant associations with the autonomic parameters in the crude and lifestyle-adjusted models but not after full adjustments (Table 5).

Generally, lifestyle variables (i.e., BMI, LTPA and smoking) and other covariates (i.e., HSCL-25, comorbidity, medication) showed stronger and more consistent associations with cardiovascular autonomic parameters than NPS or pain intensity among both sexes (Additional files 4, 5, 6, 7, 8, 9, 10 and 11).

## Discussion

The present study was a cross-sectional birth cohort study that assessed the association between musculoskeletal pains (in terms of NPS and NRS) and cardiovascular autonomic function (in terms of HR, rMSSD, SBPV, and BRS) in a large unselected sample of Northern Finns. The analyses showed some associations between NPS, pain intensity, and altered cardiovascular autonomic function, but these associations were mostly attenuated by adjustments. Thus, the results of the present study suggest that musculoskeletal pain has, at most, a limited independent association with cardiovascular autonomic function among the general Northern Finnish population.

Previous studies have investigated several groups of patients suffering from, e.g., low back pain [35, 36] and chronic pain syndromes [37], but surprisingly few large studies [18, 19] have addressed the general population with multiple pains. Importantly, results derived from large unselected samples should be of great relevance due to the high prevalence of musculoskeletal pains in the general population [38] and the subsequent economic burden on health care systems and societies [39]. Improved pain quantification methods would enhance resource allocation if they were applicable to a wide population base. As such, the present study aimed to explore the association between musculoskeletal pains and cardiovascular autonomic parameters in a large general population sample of Northern Finns.

In our data, some of the crude and partially adjusted models showed increasing NPS and pain intensity to be significantly associated with higher HR, lower rMSSD, and lower BRS, all of which point towards impaired cardiovascular autonomic function with increasing NPS and pain intensity. These findings were varyingly obtained from both sexes, such that the association between NPS and autonomic function was somewhat more consistent among women and the association between pain intensity and autonomic function among men. However, after full adjustment for lifestyle factors (BMI, LTPA, smoking) and comorbidities (HSCL-25, somatic comorbidity, medication), the associations were mostly attenuated, with only a few exceptions. The pooled-sex analyses showed significant sex*pain interactions to be related to the cardiovascular autonomic parameters.

The present results suggest that NPS is not independently associated with cardiovascular autonomic function among the general middle-aged Northern Finnish population (Primary Analysis), not even when the pain is intense and frequent (intensity ≥5 on NRS and frequency > 30 days/year; Subanalysis 1), or when it is associated with clinically relevant symptoms of depression and anxiety (HSCL-25 score ≥ 1.55; Subanalysis 2). Similarly, the overall intensity of musculoskeletal pain seems to lack an independent association with cardiovascular autonomic function in this study population. Thus, the present findings evoke two principal research questions for future studies. First, given that the confounder variables attenuated the crude associations of NPS and pain intensity with cardiovascular autonomic parameters, and also showed more systematic associations with the outcomes than NPS or pain intensity did, the question arises whether these factors may mediate the association between pain and cardiovascular autonomic function. For example, obesity and physical activity have both been associated with experiencing pain [40, 41] and ANS function [17, 42], and were systematically associated with the autonomic parameters in the present data. Due to the limitations of our study setting, future studies should aim to investigate the potential causal relationships between pain and ANS function, together with an in-depth characterization of potential mediators. Second, our results regarding sex*pain interactions suggest that the association between pain and cardiovascular autonomic function differs according to sex. This finding has also been reported in an earlier study [43], but further investigations of other datasets are needed to confirm it. In the meantime, it seems advisable to carefully assess and account for potential sex*pain interactions in future studies.

Two previous studies have investigated the association between pain and ANS parameters in large population-based (or partially population-based) samples. In a recent Norwegian study [18], 1143 individuals who suffered clinically meaningful chronic pain were compared to 5640 controls without pain in terms of several HRV and BRS parameters. After adjustment for age, sex and BMI, chronic pain was associated with reduced HRV and BRS, indicating impaired cardiovascular autonomic function. In addition to the differences between the pain variables (clinically meaningful chronic pain vs. calculation of NPS) and the modeling technique (case-control vs. continuous modeling of pain sites), also the adjustments of the Norwegian study and the present study varied considerably (age, sex, BMI vs. sex-stratification, BMI, LTPA, smoking, HSCL-25, comorbidity, medication). In the present regression models, most confounders showed clear associations with the autonomic parameters and were thus seen as relevant covariates. Similar findings regarding covariates were also reported in the Norwegian study, although it assessed fewer covariates than the present study. The present data also manifested significant sex*pain interactions which, interestingly, were tested for but not detected in the Norwegian data. It remains unclear whether the differing findings reflect differences between the studies’ definition of pain (presence of clinically meaningful chronic pain vs. NPS), comprehensiveness of the covariates, or other methodological factors.

In contrast to the Norwegian study, a Dutch study [19] found evidence for the association between widespread chronic pain and ANS dysregulation in their partially population-based sample, which consisted of both healthy individuals and patients with depressive and anxiety disorders (731 individuals reporting chronic widespread pain and 843 controls without pain). Notably, the assessed confounders were extensive (age, sex, education, alcohol use, BMI, smoking, medications, somatic and psychiatric comorbidities, physical activity, insomnia). Similar to the present study, the Dutch study detected several significant associations between pain and the ANS variables in the crude models, but not after controlling for the confounders. The authors stated that previous studies have been potentially hampered by insufficient control of potential confounders, small sample sizes and the use of patient samples instead of population-based approaches. Prospective, longitudinal studies utilizing large representative samples are needed to further address these considerations.

The main strength of the present study is its large general population sample with extensive data on pain, lifestyle and health, allowing the assessment of NPS, pain intensity, and cardiovascular autonomic function, while accounting for numerous confounding factors. In comparison with patient samples, the population-based setting of the present study increased the generalizability of its results. Furthermore, the present study was designed to address musculoskeletal pains at several anatomical locations instead of focusing on a certain pain site, indicating that the present approach was comprehensive, and took into account the possible widespread nature of pain. The present study was also able to assess several cardiovascular autonomic parameters, addressing both HRV and BRS.

A major limitation of this study is its cross-sectional setting. The outcomes (i.e., cardiovascular autonomic parameters) and primary predictors (i.e., NPS/NRS) were only recorded at the most recent follow-up of the cohort study, i.e., at the age of 46. Despite the large, well-characterized sample, the lack of longitudinal data on pain may have reduced the strength of the variable and may thus partially explain the lack of consistent association between pain and cardiovascular autonomic function. The pain data may also be limited because the pain survey enquired musculoskeletal pains over a period of 12 months. This may have increased the likelihood of recall bias, and also decreased the temporal accordance between the exposure (i.e., pain) and outcome (i.e., cardiovascular autonomic function). Unfortunately, the study subjects’ musculoskeletal pain status was not enquired on the clinical examination day when the autonomic parameters were obtained due to NFBC1966’s data collection protocol. However, it is notable that many crude models demonstrated clear associations between NPS, pain intensity, and cardiovascular autonomic parameters, and these associations were only attenuated after adjustments. As such, these findings suggest that the lack of association is primarily explained by the presence of confounding factors and not by the limitations of the pain data themselves. In the calculation of NPS, each anatomical location was weighted equally due to the lack of more accurate calculation protocols. However, NPS is a widely used measure of musculoskeletal pain [21] and has been associated with, e.g., overall health and sleep quality in the general population [22]. The linear relationship between NPS and cardiovascular autonomic parameters was only assumed after no other relationship patterns were detected. With regard to the outcomes, we acknowledge that our ANS parameters were indicators of ANS-based cardiovascular function, as opposed to other aspects of ANS function. Thus, the present conclusions primarily concern cardiovascular autonomic function, leaving the more peripheral ANS to be explored in future studies.

## Conclusions

This cross-sectional population-based study of Northern Finns found some evidence of a relationship between NPS, pain intensity, and cardiovascular autonomic dysfunction among both sexes. However, these associations were mostly attenuated after adjustment for lifestyle factors and comorbidities. Thus, the present results suggest that musculoskeletal pain has, at most, a limited independent association with cardiovascular autonomic function in the general Northern Finnish population, even when the pain is intense and frequent, or associated with clinically relevant symptoms of depression and anxiety. Future studies should confirm the present findings in other general population samples with longitudinal designs, and take into account the potential confounders and sex interactions that this study revealed. If a causal relationship between musculoskeletal pain and ANS is detected, the potential mediators should also be characterized.

## Additional files

Additional file 1: Summary of outcome variables. (DOCX 27 kb)

Additional file 2: Predictor and covariate variables of the regression models. (DOCX 24 kb)

Additional file 3: Construction of regression models. (DOCX 27 kb)

Additional file 4: Primary Analysis, men. (DOCX 52 kb)

Additional file 5: Primary Analysis, women. (DOCX 51 kb)

Additional file 6: Subanalysis 1, men. (DOCX 50 kb)

Additional file 7: Subanalysis 1, women. (DOCX 50 kb)

Additional file 8: Subanalysis 2, men. (DOCX 48 kb)

Additional file 9: Subanalysis 2, women. (DOCX 48. kb)

Additional file 10: Subanalysis 3, men. (DOCX 52 kb)

Additional file 11: Subanalysis 3, women. (DOCX 52 kb)

## Abbreviations

ANS: Autonomic nervous system
BMI: Body mass index
BRS: Baroreflex sensitivity
CI: Confidence interval
HR: Heart rate
HRV: Heart rate variability
HSCL-25: Hopkins Symptom Checklist-25
LTPA: Leisure-time physical activity
NPS: Number of pain sites
NRS: Numerical Rating Scale
P: *P* value
rMSSD: Root mean square of the successive differences in R-R intervals
SBPV: Systolic blood pressure variability
β: Beta coefficient
