# Supplementary material for: Musculoskeletal pains and cardiovascular autonomic function in the general Northern Finnish population
Source: BMC Musculoskelet Disord. 2019 Jan 31;20:45. doi: 10.1186/s12891-019-2426-2 (PMC6357438; doi:10.1186/s12891-019-2426-2)
Supplement: Supplementary file 1 — Summary of outcome variables. (DOCX 27 kb) [file 12891_2019_2426_MOESM1_ESM.docx]

**Additional file 1**. Summary of outcome variables. Data acquisition and processing protocols are described under Methods.

| Variable | Abbreviation | Unit | Definition | Origin of data | Transformation to Gaussian |
| --- | --- | --- | --- | --- | --- |
| Heart rate (seated and standing) | HR | bpm | Mean of R-R intervals | HRV data (obtained at all follow-up units) | Not needed |
| Root mean square of the successive differences in R-R intervals (seated and standing) | rMSSD | ms | Root mean square of the successive differences in R-R intervals | HRV data (obtained at all follow-up units) | Natural logarithm |
| Systolic blood pressure variability (seated and standing) | SBPV | mmHg^2^ | Short-term blood pressure variability | BRS data (obtained at Oulu follow-up unit only) | Natural logarithm |
| Cross-spectral baroreflex sensitivity (seated and standing) | BRS | ms/mmHg | R-R interval response relative to change in systolic blood pressure | BRS data (obtained at Oulu follow-up unit only) | Natural logarithm |
