# Supplementary material for: Musculoskeletal pains and cardiovascular autonomic function in the general Northern Finnish population
Source: BMC Musculoskelet Disord. 2019 Jan 31;20:45. doi: 10.1186/s12891-019-2426-2 (PMC6357438; doi:10.1186/s12891-019-2426-2)
Supplement: Supplementary file 2 — Predictor and covariate variables of the regression models. (DOCX 24 kb) [file 12891_2019_2426_MOESM2_ESM.docx]

**Additional file 2**. Predictor and covariate variables of the regression models.

| Variable | Abbreviation | Unit | Continuous/ categorical | Coding of categorical variables |
| --- | --- | --- | --- | --- |
| Number of pain sites | NPS | - | Continuous | - |
| Pain intensity according to Numerical Rating Scale (0–10) | NRS | - | Continuous | - |
| Body mass index | BMI | kg/m^2^ | Continuous | - |
| Leisure-time physical activity | LTPA | - | Categorical | 0 = < 1/week (reference)  1 = 1/week  2 = 2–3/week  3 = ≥ 4/week |
| Smoking | - | - | Categorical | 0 = Non-smoker (reference)  1 = Former smoker  2 = Current smoker |
| Hopkins Symptom Checklist-25 | HSCL-25 | Score | Continuous | - |
| Comorbidities associated with ANS function | - | - | Categorical | 0 = No (reference)  1 = Yes |
| Mediations associated with ANS function | - | - | Categorical | 0 = No (reference)  1 = Yes |

ANS = Autonomic nervous system
