# Supplementary material for: Musculoskeletal pains and cardiovascular autonomic function in the general Northern Finnish population
Source: BMC Musculoskelet Disord. 2019 Jan 31;20:45. doi: 10.1186/s12891-019-2426-2 (PMC6357438; doi:10.1186/s12891-019-2426-2)
Supplement: Supplementary file 3 — Construction of regression models. (DOCX 27 kb) [file 12891_2019_2426_MOESM3_ESM.docx]

**Additional file 3**. Construction of regression models. All models were run for each outcome separately.

| Model specification | Primary predictor | Covariates | Stratification |
| --- | --- | --- | --- |
| I (crude) | NPS/NRS* | - | Sex |
| III (adjusted for lifestyle) | NPS/NRS* | BMI, LTPA, smoking | Sex |
| IV (adjusted for comorbidities) | NPS/NRS* | HSCL-25**, comorbidities, medications | Sex |
| V (adjusted for lifestyle and comorbidities) | NPS/NRS* | BMI, LTPA, smoking, HSCL-25**, comorbidities, medications | Sex |

*NPS was the primary predictor in Primary Analysis, Subanalysis 1 and Subanalysis 2. NRS was the primary predictor in Subanalysis 3.

**Not implemented in Subanalysis 2 due to the use of HSCL-25 as inclusion criterion.

BMI = Body mass index, HSCL-25 = Hopkins Symptom Checklist-25, LTPA = Leisure-time physical activity, NPS = Number of pain sites, NRS = Pain intensity according to Numerical Rating Scale.
