# Supplementary material for: Musculoskeletal pains and cardiovascular autonomic function in the general Northern Finnish population
Source: BMC Musculoskelet Disord. 2019 Jan 31;20:45. doi: 10.1186/s12891-019-2426-2 (PMC6357438; doi:10.1186/s12891-019-2426-2)
Supplement: Supplementary file 4 — Primary Analysis, men. (DOCX 52 kb) [file 12891_2019_2426_MOESM4_ESM.docx]

**Additional file 4.** Primary Analysis, men. Complete linear regression models for the association between number of pain sites (NPS) and cardiovascular autonomic function (HR, rMSSD, SBPV, BRS) among men (for HR and rMSSD, n = 1813; for SBPV and BRS, n = 919). Variable coding, reference groups and model construction are presented in Additional files 1–3.

| Variables | Model I | | |  | Model II | | |  | Model III | | |  | Model IV | | |
| --- | --- | --- | --- | --- | --- | --- | --- | --- | --- | --- | --- | --- | --- | --- | --- |
|  | β [95% CI] |  | P |  | β [95% CI] |  | P |  | β [95% CI] |  | P |  | β [95% CI] |  | P |
| **Outcome: HR, seated** |  |  |  |  |  |  |  |  |  |  |  |  |  |  |  |
| NPS | 0.125 [-0.140; 0.391] |  | 0.355 |  | -0.117 [-0.374; 0.139] |  | 0.370 |  | -0.016 [-0.288; 0.256] |  | 0.906 |  | -0.184 [-0.447; 0.079] |  | 0.169 |
| BMI |  |  |  |  | 0.568 [0.436; 0.701] |  | < 0.001 |  |  |  |  |  | 0.565 [0.427; 0.702] |  | < 0.001 |
| LTPA = 1 |  |  |  |  | -2.223 [-3.759; -0.688] |  | 0.005 |  |  |  |  |  | -2.086 [-3.624; -0.547] |  | 0.008 |
| LTPA = 2 |  |  |  |  | -4.333 [-5.691; -2.975] |  | < 0.001 |  |  |  |  |  | -4.186 [-5.550; -2.822] |  | < 0.001 |
| LTPA = 3 |  |  |  |  | -6.107 [-7.853; -4.362] |  | < 0.001 |  |  |  |  |  | -5.921 [-7.674; -4.168] |  | < 0.001 |
| Smoking = 1 |  |  |  |  | 0.200 [-1.034; 1.434] |  | 0.751 |  |  |  |  |  | 0.120 [-1.117; 1.356] |  | 0.849 |
| Smoking = 2 |  |  |  |  | 3.064 [1.642; 4.486] |  | < 0.001 |  |  |  |  |  | 2.837 [1.404; 4.269] |  | < 0.001 |
| HSCL-25 |  |  |  |  |  |  |  |  | 3.798 [1.899; 5.696] |  | < 0.001 |  | 2.203 [0.355; 4.051] |  | 0.019 |
| Comorbidity = 1 |  |  |  |  |  |  |  |  | 2.749 [0.800; 4.697] |  | 0.006 |  | 0.436 [-1.478; 2.350] |  | 0.655 |
| Medication = 1 |  |  |  |  |  |  |  |  | 0.619 [-0.969; 2.207] |  | 0.445 |  | -0.559 [-2.102; 0.984] |  | 0.478 |
|  |  |  |  |  |  |  |  |  |  |  |  |  |  |  |  |
| **Outcome: HR, standing** |  |  |  |  |  |  |  |  |  |  |  |  |  |  |  |
| NPS | 0.188 [-0.105; 0.481] | | 0.208 |  | -0.015 [-0.304; 0.274] |  | 0.920 |  | 0.036 [-0.265; 0.337] |  | 0.815 |  | -0.099 [-0.395; 0.197] |  | 0.512 |
| BMI |  | |  |  | 0.341 [0.192; 0.490] |  | < 0.001 |  |  |  |  |  | 0.359 [0.204; 0.514] |  | < 0.001 |
| LTPA = 1 |  |  |  |  | -2.150 [-3.880; -0.420] |  | 0.015 |  |  |  |  |  | -1.955 [-3.687; -0.224] |  | 0.027 |
| LTPA = 2 |  |  |  |  | -4.413 [-5.943; -2.883] |  | < 0.001 |  |  |  |  |  | -4.268 [-5.803; -2.733] |  | < 0.001 |
| LTPA = 3 |  | |  |  | -6.218 [-8.184; -4.251] |  | < 0.001 |  |  |  |  |  | -6.033 [-8.005; -4.060] |  | < 0.001 |
| Smoking = 1 |  | |  |  | 0.286 [-1.104; 1.677] |  | 0.686 |  |  |  |  |  | 0.213 [-1.178; 1.604] |  | 0.764 |
| Smoking = 2 |  | |  |  | 3.160 [1.558; 4.762] |  | < 0.001 |  |  |  |  |  | 2.866 [1.254; 4.478] |  | 0.001 |
| HSCL-25 |  | |  |  |  |  |  |  | 4.359 [2.260; 6.458] |  | < 0.001 |  | 2.819 [0.739; 4.898] |  | 0.008 |
| Comorbidity = 1 |  | |  |  |  |  |  |  | 2.010 [-0.144; 4.165] |  | 0.067 |  | 0.296 [-1.857; 2.449] |  | 0.788 |
| Medication = 1 |  | |  |  |  |  |  |  | -0.918 [-2.674; 0.838] |  | 0.305 |  | -1.767 [-3.504; -0.031] |  | 0.046 |
|  |  | |  |  |  |  |  |  |  |  |  |  |  |  |  |
| **Outcome: rMSSD, seated** |  | |  |  |  |  |  |  |  |  |  |  |  |  |  |
| NPS | -0.010 [-0.024; 0.004] | | 0.142 |  | 0.002 [-0.011; 0.016] |  | 0.755 |  | -0.005 [-0.019; 0.010] |  | 0.516 |  | 0.004 [-0.010; 0.018] |  | 0.546 |
| BMI |  | |  |  | -0.032 [-0.039; -0.025] |  | < 0.001 |  |  |  |  |  | -0.031 [-0.039; -0.024] |  | < 0.001 |
| LTPA = 1 |  | |  |  | 0.113 [0.032; 0.194] |  | 0.007 |  |  |  |  |  | 0.110 [0.028; 0.191] |  | 0.008 |
| LTPA = 2 |  | |  |  | 0.181 [0.110; 0.253] |  | < 0.001 |  |  |  |  |  | 0.176 [0.104; 0.248] |  | < 0.001 |
| LTPA = 3 |  | |  |  | 0.293 [0.201; 0.386] |  | < 0.001 |  |  |  |  |  | 0.286 [0.193; 0.379] |  | < 0.001 |
| Smoking = 1 |  | |  |  | -0.012 [-0.077; 0.053] |  | 0.718 |  |  |  |  |  | -0.008 [-0.074; 0.057] |  | 0.800 |
| Smoking = 2 |  | |  |  | -0.152 [-0.228; -0.077] |  | < 0.001 |  |  |  |  |  | -0.147 [-0.223; -0.071] |  | < 0.001 |
| HSCL-25 |  | |  |  |  |  |  |  | -0.142 [-0.242; -0.042] |  | 0.006 |  | 0.064 [-0.162; 0.034] |  | 0.200 |
| Comorbidity = 1 |  | |  |  |  |  |  |  | -0.117 [-0.220; -0.014] |  | 0.026 |  | 0.006 [-0095; 0.107] |  | 0.909 |
| Medication = 1 |  | |  |  |  |  |  |  | -0.099 [-0.183; -0.015] |  | 0.021 |  | -0.037 [-0.119; 0.045] |  | 0.377 |
|  |  | |  |  |  |  |  |  |  |  |  |  |  |  |  |
| **Outcome: rMSSD, standing** |  | |  |  |  |  |  |  |  |  |  |  |  |  |  |
| NPS | -0.011 [-0.025; 0.003] | | 0.130 |  | 0.000 [-0.014; 0.014] |  | 0.986 |  | -0.005 [-0.019; 0.009] |  | 0.502 |  | 0.002 [-0.012; 0.016] |  | 0.749 |
| BMI |  | |  |  | -0.023 [-0.030; -0.016] |  | < 0.001 |  |  |  |  |  | -0.021 [-0.028; -0.013] |  | < 0.001 |
| LTPA = 1 |  | |  |  | 0.094 [0.012; 0.176] |  | 0.024 |  |  |  |  |  | 0.091 [0.009; 0.173] |  | 0.030 |
| LTPA = 2 |  | |  |  | 0.144 [0.072; 0.217] |  | < 0.001 |  |  |  |  |  | 0.136 [0.063; 0.208] |  | < 0.001 |
| LTPA = 3 |  | |  |  | 0.246 [0.153; 0.339] |  | < 0.001 |  |  |  |  |  | 0.235 [0.142; 0.329] |  | < 0.001 |
| Smoking = 1 |  | |  |  | 0.014 [-0.052; 0.080] |  | 0.676 |  |  |  |  |  | 0.019 [-0.047; 0.085] |  | 0.567 |
| Smoking = 2 |  | |  |  | -0.174 [-0.250; -0.098] |  | < 0.001 |  |  |  |  |  | -0.166 [-0.242; -0.090] |  | < 0.001 |
| HSCL-25 |  | |  |  |  |  |  |  | -0.144 [-0.243; -0.045] |  | 0.005 |  | -0.076 [-0.174; 0.023] |  | 0.132 |
| Comorbidity = 1 |  | |  |  |  |  |  |  | -0.138 [-0.240; -0.036] |  | 0.008 |  | -0.049 [-0.151; 0.053] |  | 0.348 |
| Medication = 1 |  | |  |  |  |  |  |  | -0.103 [-0.186; -0.019] |  | 0.016 |  | -0.064 [-0.147; 0.018] |  | 0.124 |
|  |  | |  |  |  |  |  |  |  |  |  |  |  |  |  |
| **Outcome: SBPV, seated** |  | |  |  |  |  |  |  |  |  |  |  |  |  |  |
| NPS | -0.001 [-0.026; 0.024] | | 0.946 |  | 0.000 [-0.025; 0.026] |  | 0.970 |  | 0.006 [-0.020; 0.032] |  | 0.661 |  | 0.005 [-0.021; 0.032] |  | 0.691 |
| BMI |  | |  |  | 0.000 [-0.014; 0.014] |  | 0.989 |  |  |  |  |  | 0.000 [-0.021; 0.032] |  | 0.966 |
| LTPA = 1 |  | |  |  | 0.005 [-0.147; 0.157] |  | 0.951 |  |  |  |  |  | 0.004 [-0.148; 0.157] |  | 0.954 |
| LTPA = 2 |  | |  |  | 0.005 [-0.128; 0.139] |  | 0.938 |  |  |  |  |  | -0.004 [-0.139; 0.130] |  | 0.950 |
| LTPA = 3 |  | |  |  | -0.151 [-0.321; 0.019] |  | 0.081 |  |  |  |  |  | -0.158 [-0.329; 0.013] |  | 0.069 |
| Smoking = 1 |  | |  |  | -0.037 [-0.159; 0.084] |  | 0.549 |  |  |  |  |  | -0.029 [-0.151; 0.093] |  | 0.644 |
| Smoking = 2 |  | |  |  | -0.232 [-0.372; -0.092] |  | 0.001 |  |  |  |  |  | -0.222 [-0.364; -0.080] |  | 0.002 |
| HSCL-25 |  | |  |  |  |  |  |  | -0.146 [-0.326; 0.033] |  | 0.109 |  | -0.108 [-0.290; 0.075] |  | 0.247 |
| Comorbidity = 1 |  | |  |  |  |  |  |  | 0.092 [-0.106; 0.290] |  | 0.361 |  | 0.094 {-0.107; 0.294] |  | 0.294 |
| Medication = 1 |  | |  |  |  |  |  |  | -0.074 [-0.225; 0.077] |  | 0.337 |  | -0.086 [-0.239; 0.067] |  | 0.271 |
|  |  | |  |  |  |  |  |  |  |  |  |  |  |  |  |
| **Outcome: SBPV, standing** |  | |  |  |  |  |  |  |  |  |  |  |  |  |  |
| NPS | -0.014 [-0.039; 0.011] | | 0.280 |  | -0.012 [-0.038; 0.014] |  | 0.361 |  | 0.001 [-0.025; 0.027] |  | 0.937 |  | -0.001 [-0.027; 0.026] |  | 0.957 |
| BMI |  | |  |  | 0.010 [-0.004; 0.024] |  | 0.159 |  |  |  |  |  | 0.015 [0.001; 0.029] |  | 0.039 |
| LTPA = 1 |  | |  |  | 0.032 [-1.22; 0.185] |  | 0.686 |  |  |  |  |  | 0.026 [-0.126; 0.178] |  | 0.737 |
| LTPA = 2 |  | |  |  | 0.052 [-0.082; 0.187] |  | 0.447 |  |  |  |  |  | 0.019 [-0.115; 0.153] |  | 0.783 |
| LTPA = 3 |  | |  |  | -0.083 [-0.254; 0.088] |  | 0.341 |  |  |  |  |  | -0.115 [-0.285; 0.055] |  | 0.184 |
| Smoking = 1 |  | |  |  | -0.035 [-0.157; 0.088] |  | 0.580 |  |  |  |  |  | -0.007 [-0.192; 0.114] |  | 0.906 |
| Smoking = 2 |  | |  |  | -0.300 [-0.442; -0.159] |  | < 0.001 |  |  |  |  |  | -0.263 [-0.404; -0.121] |  | < 0.001 |
| HSCL-25 |  | |  |  |  |  |  |  | -0.337 [-0.517; -0.157] |  | < 0.001 |  | -0.287 [-0.469; -0.106] |  | 0.002 |
| Comorbidity = 1 |  | |  |  |  |  |  |  | -0.006 [-0.204; 0.193] |  | 0.956 |  | -0.034 [-0.233; 0.166] |  | 0.742 |
| Medication = 1 |  | |  |  |  |  |  |  | -0.220 [-0.371; -0.069] |  | 0.004 |  | -0.255 [-0.408; -0.103] |  | 0.001 |
|  |  | |  |  |  |  |  |  |  |  |  |  |  |  |  |
| **Outcome: BRS, seated** |  | |  |  |  |  |  |  |  |  |  |  |  |  |  |
| NPS | -0.002 [-0.019; 0.015] | | 0.820 |  | 0.011 [-0.005; 0.027] |  | 0.189 |  | 0.003 [-0.015; 0.020] |  | 0.753 |  | 0.013 [-0.004; 0.029] |  | 0.136 |
| BMI |  | |  |  | -0.036 [-0.045; -0.027] |  | < 0.001 |  |  |  |  |  | -0.034 [-0.043; -0.025] |  | < 0.001 |
| LTPA = 1 |  | |  |  | 0.082 [-0.014; 0.178] |  | 0.096 |  |  |  |  |  | 0.079 [-0.018; 0.176] |  | 0.109 |
| LTPA = 2 |  | |  |  | 0.099 [0.015; 0.184] |  | 0.022 |  |  |  |  |  | 0.092 [0.007; 0.177] |  | 0.035 |
| LTPA = 3 |  | |  |  | 0.259 [0.152; 0.367] |  | < 0.001 |  |  |  |  |  | 0.251 [0.143; 0.359] |  | < 0.001 |
| Smoking = 1 |  | |  |  | -0.072 [-0.148; 0.005] |  | 0.068 |  |  |  |  |  | -0.066 [-0.143; 0.011] |  | 0.094 |
| Smoking = 2 |  | |  |  | -0.120 [-0.209; -0.032] |  | 0.008 |  |  |  |  |  | -0.111 [-0.201; -0.021] |  | 0.016 |
| HSCL-25 |  | |  |  |  |  |  |  | -0.113 [-0.231; 0.006] |  | 0.062 |  | -0.058 [-0.174; 0.057] |  | 0.320 |
| Comorbidity = 1 |  | |  |  |  |  |  |  | -0.165 [-0.296; -0.035] |  | 0.013 |  | -0.059 [-0.185; 0.068] |  | 0.364 |
| Medication = 1 |  | |  |  |  |  |  |  | -0.104 [-0.024; -0.005] |  | 0.040 |  | -0.041 [-0.138; 0.055] |  | 0.403 |
|  |  | |  |  |  |  |  |  |  |  |  |  |  |  |  |
| **Outcome: BRS, standing** |  | |  |  |  |  |  |  |  |  |  |  |  |  |  |
| NPS | -0.001 [-0.019; 0.016] | | 0.873 |  | 0.011 [-0.006; 0.028] |  | 0.208 |  | 0.003 [-0.015; 0.021] |  | 0.718 |  | 0.013 [-0.005; 0.030] |  | 0.149 |
| BMI |  | |  |  | -0.036 [-0.045; -0.027] |  | < 0.001 |  |  |  |  |  | -0.034 [-0.044; -0.025] |  | < 0.001 |
| LTPA = 1 |  | |  |  | 0.060 [-0.040; 0.161] |  | 0.241 |  |  |  |  |  | 0.057 [-0.044; 0.158] |  | 0.296 |
| LTPA = 2 |  | |  |  | 0.074 [-0.015; 0.162] |  | 0.102 |  |  |  |  |  | 0.067 [-0.022; 0.155] |  | 0.142 |
| LTPA = 3 |  | |  |  | 0.210 [0.098; 0.322] |  | < 0.001 |  |  |  |  |  | 0.202 [0.089; 0.315] |  | < 0.001 |
| Smoking = 1 |  | |  |  | -0.024 [-0.104; 0.057] |  | 0.561 |  |  |  |  |  | -0.019 [-0.099; 0.062] |  | 0.651 |
| Smoking = 2 |  | |  |  | -0.144 [-0.236; -0.051] |  | 0.002 |  |  |  |  |  | -0.134 [-0.228; -0.040] |  | 0.005 |
| HSCL-25 |  | |  |  |  |  |  |  | -0.114 [-0.237; 0.009] |  | 0.070 |  | -0.061 [-0.181; 0.060] |  | 0.321 |
| Comorbidity = 1 |  | |  |  |  |  |  |  | -0.156 [-0.291; -0.020] |  | 0.024 |  | -0.050 [-0.183; 0.082] |  | 0.456 |
| Medication = 1 |  | |  |  |  |  |  |  | -0.088 [-0.191; 0.015] |  | 0.094 |  | -0.033 [-0.133; 0.068] |  | 0.528 |
