# Supplementary material for: Musculoskeletal pains and cardiovascular autonomic function in the general Northern Finnish population
Source: BMC Musculoskelet Disord. 2019 Jan 31;20:45. doi: 10.1186/s12891-019-2426-2 (PMC6357438; doi:10.1186/s12891-019-2426-2)
Supplement: Supplementary file 5 — Primary Analysis, women. (DOCX 51 kb) [file 12891_2019_2426_MOESM5_ESM.docx]

**Additional file 5.** Primary Analysis, women. Complete linear regression models for the association between number of pain sites (NPS) and cardiovascular autonomic function (HR, rMSSD, SBPV, BRS) among women (for HR and rMSSD, n = 2373; for SBPV and BRS, n = 1112). Variable coding, reference groups and model construction are presented in Additional files 1–3.

| Variables | Model I | | |  | Model II | | |  | Model III | | |  | Model IV | | |
| --- | --- | --- | --- | --- | --- | --- | --- | --- | --- | --- | --- | --- | --- | --- | --- |
|  | β [95% CI] |  | P |  | β [95% CI] |  | P |  | β [95% CI] |  | P |  | β [95% CI] |  | P |
| **Outcome: HR, seated** |  |  |  |  |  |  |  |  |  |  |  |  |  |  |  |
| NPS | 0.318 [0.119; 0.516] |  | 0.002 |  | 0.179 [-0.017; 0.375] |  | 0.073 |  | 0.230 [0.024; 0.437] |  | 0.029 |  | 0.147 [-0.055; 0.350] |  | 0.154 |
| BMI |  |  |  |  | 0.360 [0.275; 0.445] |  | < 0.001 |  |  |  |  |  | 0.345 [0.257; 0.433] |  | < 0.001 |
| LTPA = 1 |  |  |  |  | -0.554 [-1.789; 0.681] |  | 0.379 |  |  |  |  |  | -0.482 [-1.718; 0.753] |  | 0.444 |
| LTPA = 2 |  |  |  |  | -1.757 [-2.882; -0.631] |  | 0.002 |  |  |  |  |  | -1.677 [-2.806; -0.548] |  | 0.004 |
| LTPA = 3 |  |  |  |  | -3.814 [-5.180; -2.447] |  | < 0.001 |  |  |  |  |  | -3.757 [-5.128; -2.386] |  | < 0.001 |
| Smoking = 1 |  |  |  |  | -1.726 [-2.720; -0.733] |  | 0.001 |  |  |  |  |  | -1.725 [-2.720; -0.730] |  | 0.001 |
| Smoking = 2 |  |  |  |  | -0.673 [-1.837; 0.491] |  | 0.257 |  |  |  |  |  | -0.734 [-1.902; 0.435] |  | 0.218 |
| HSCL-25 |  |  |  |  |  |  |  |  | 0.609 [-0.716; 1.935] |  | 0.367 |  | 0.261 [-10.050; 1.572] |  | 0.697 |
| Comorbidity = 1 |  |  |  |  |  |  |  |  | 3.285 [1.755; 4.814] |  | < 0.001 |  | 2.242 [0.730; 3.754] |  | 0.004 |
| Medication = 1 |  |  |  |  |  |  |  |  | 0.747 [-0.472; 1.966] |  | 0.230 |  | -0.390 [-1.608; 0.828] |  | 0.530 |
|  |  |  |  |  |  |  |  |  |  |  |  |  |  |  |  |
| **Outcome: HR, standing** |  |  |  |  |  |  |  |  |  |  |  |  |  |  |  |
| NPS | 0.299 [0.066; 0.531] | | 0.012 |  | 0.203 [-0.029; 0.435] |  | 0.086 |  | 0.256 [0.014; 0.498] |  | 0.038 |  | 0.196 [-0.044; 0.436] |  | 0.110 |
| BMI |  | |  |  | 0.241 [0.140; 0.342] |  | < 0.001 |  |  |  |  |  | 0.242 [0.138; 0.346] |  | < 0.001 |
| LTPA = 1 |  |  |  |  | -0.760 [-2.223; 0.702] |  | 0.308 |  |  |  |  |  | -0.736 [-2.200; 0.729] |  | 0.325 |
| LTPA = 2 |  |  |  |  | -2.120 [-3.453; -0.786] |  | 0.002 |  |  |  |  |  | -2.064 [-3.403; -0.726] |  | 0.003 |
| LTPA = 3 |  | |  |  | -4.264 [-5.882; -2.645] |  | < 0.001 |  |  |  |  |  | -4.273 [-5.899; -2.647] |  | < 0.001 |
| Smoking = 1 |  | |  |  | -1.761 [-2.937; -0.584] |  | 0.003 |  |  |  |  |  | -1.751 [-2.930; -0.571] |  | 0.004 |
| Smoking = 2 |  | |  |  | -1.872 [-3.251; -0.493] |  | 0.008 |  |  |  |  |  | -1.882 [-3.267; -0.496] |  | 0.008 |
| HSCL-25 |  | |  |  |  |  |  |  | 0.272 [-1.283; 1.828] |  | 0.731 |  | 0.022 [-1.532; 1.576] |  | 0.978 |
| Comorbidity = 1 |  | |  |  |  |  |  |  | 2.262 [0.467; 4.057] |  | 0.014 |  | 1.505 [-0.288; 3.298] |  | 0.100 |
| Medication = 1 |  | |  |  |  |  |  |  | -0.151 [-1.581; 1.280] |  | 0.836 |  | -0.991 [-2.436; 0.453] |  | 0.179 |
|  |  | |  |  |  |  |  |  |  |  |  |  |  |  |  |
| **Outcome: rMSSD, seated** |  | |  |  |  |  |  |  |  |  |  |  |  |  |  |
| NPS | -0.020 [-0.032; -0.009] | | < 0.001 |  | -0.011 [-0.022; -0.000] |  | 0.049 |  | -0.011 [-0.023; 0.001] |  | 0.071 |  | -0.006 [-0.017; 0.006] |  | 0.323 |
| BMI |  | |  |  | -0.024 [-0.029; -0.019] |  | < 0.001 |  |  |  |  |  | -0.021 [-0.026; -0.016] |  | < 0.001 |
| LTPA = 1 |  | |  |  | 0.004 [-0.067; 0.074] |  | 0.915 |  |  |  |  |  | -0.005 [-0.075; 0.065] |  | 0.888 |
| LTPA = 2 |  | |  |  | 0.056 [-0.008; 0.120] |  | 0.088 |  |  |  |  |  | 0.049 [-0.015; 0.113] |  | 0.137 |
| LTPA = 3 |  | |  |  | 0.153 [0.075; 0.231] |  | < 0.001 |  |  |  |  |  | 0.139 [0.061; 0.217] |  | 0.001 |
| Smoking = 1 |  | |  |  | 0.041 [-0.016; 0.098] |  | 0.155 |  |  |  |  |  | 0.044 [-0.013; 0.101] |  | 0.127 |
| Smoking = 2 |  | |  |  | -0.020 [-0.086; 0.047] |  | 0.563 |  |  |  |  |  | -0.008 [-0.075; 0.058] |  | 0.805 |
| HSCL-25 |  | |  |  |  |  |  |  | -0.090 [-0.166; -0.015] |  | 0.019 |  | -0.070 [-0.145; 0.005] |  | 0.067 |
| Comorbidity = 1 |  | |  |  |  |  |  |  | -0.213 [-0.300; -0.126] |  | < 0.001 |  | -0.153 [-0.239; -0.067] |  | 0.001 |
| Medication = 1 |  | |  |  |  |  |  |  | -0.146 [-0.215; -0.077] |  | < 0.001 |  | -0.080 [-0.149; -0.010] |  | 0.025 |
|  |  | |  |  |  |  |  |  |  |  |  |  |  |  |  |
| **Outcome: rMSSD, standing** |  | |  |  |  |  |  |  |  |  |  |  |  |  |  |
| NPS | -0.016 [-0.027; -0.004] | | 0.008 |  | -0.009 [-0.020; 0.003] |  | 0.136 |  | -0.008 [-0.020; 0.004] |  | 0.195 |  | -0.004 [-0.016; 0.008] |  | 0.472 |
| BMI |  | |  |  | -0.016 [-0.021; -0.011] |  | < 0.001 |  |  |  |  |  | -0.014 [-0.019; -0.009] |  | < 0.001 |
| LTPA = 1 |  | |  |  | 0.010 [-0.063; 0.082] |  | 0.796 |  |  |  |  |  | 0.002 [-0.071; 0.074] |  | 0.965 |
| LTPA = 2 |  | |  |  | 0.063 [-0.003; 0.130] |  | 0.061 |  |  |  |  |  | 0.058 [-0.009; 0.124] |  | 0.089 |
| LTPA = 3 |  | |  |  | 0.147 [0.066; 0.228] |  | < 0.001 |  |  |  |  |  | 0.136 [0.055; 0.217] |  | 0.001 |
| Smoking = 1 |  | |  |  | 0.028 [-0.030; 0.087] |  | 0.344 |  |  |  |  |  | 0.030 [-0.029; 0.089] |  | 0.314 |
| Smoking = 2 |  | |  |  | -0.030 [-0.099; 0.038] |  | 0.386 |  |  |  |  |  | -0.021 [-0.090; 0.048] |  | 0.544 |
| HSCL-25 |  | |  |  |  |  |  |  | -0.070 [-0.147; 0.007] |  | 0.076 |  | -0.049 [-0.126; 0.028] |  | 0.214 |
| Comorbidity = 1 |  | |  |  |  |  |  |  | -0.189 [-0.278; -0.101] |  | < 0.001 |  | -0.147 [-0.236; -0.058] |  | 0.001 |
| Medication = 1 |  | |  |  |  |  |  |  | -0.115 [-0.185; -0.044] |  | 0.002 |  | -0.069 [-0.140; 0.003] |  | 0.060 |
|  |  | |  |  |  |  |  |  |  |  |  |  |  |  |  |
| **Outcome: SBPV, seated** |  | |  |  |  |  |  |  |  |  |  |  |  |  |  |
| NPS | -0.001 [-0.024; 0.022] | | 0.924 |  | 0.000 [-0.023; 0.023] |  | 0.991 |  | 0.003 [-0.021; 0.027] |  | 0.797 |  | 0.003 [-0.021; 0.027] |  | 0.819 |
| BMI |  | |  |  | 0.005 [-0.005; 0.014] |  | 0.364 |  |  |  |  |  | 0.006 [-0.004; 0.016] |  | 0.233 |
| LTPA = 1 |  | |  |  | 0.005 [-0.141; 0.150] |  | 0.949 |  |  |  |  |  | -0.005 [-0.152; 0.141] |  | 0.942 |
| LTPA = 2 |  | |  |  | 0.053 [-0.080; 0.186] |  | 0.434 |  |  |  |  |  | 0.047 [-0.086; 0.181] |  | 0.487 |
| LTPA = 3 |  | |  |  | -0.005 [-0.165; 0.155] |  | 0.948 |  |  |  |  |  | -0.016 [-0.177; 0.146] |  | 0.849 |
| Smoking = 1 |  | |  |  | -0.084 [-0.204; 0.035] |  | 0.167 |  |  |  |  |  | -0.081 [-0.202; 0.039] |  | 0.184 |
| Smoking = 2 |  | |  |  | 0.187 [-0.323; -0.050] |  | 0.007 |  |  |  |  |  | -0.181 [-0.318; -0.043] |  | 0.010 |
| HSCL-25 |  | |  |  |  |  |  |  | -0.060 [-0.216; 0.097] |  | 0.454 |  | -0.028 [-0.187; 0.131] |  | 0.727 |
| Comorbidity = 1 |  | |  |  |  |  |  |  | -0.045 [-0.212; 0.121] |  | 0.593 |  | -0.053 [-0.223; 0.116] |  | 0.538 |
| Medication = 1 |  | |  |  |  |  |  |  | -0.050 [-0.188; 0.088] |  | 0.475 |  | -0.067 [-0.208; 0.074] |  | 0.349 |
|  |  | |  |  |  |  |  |  |  |  |  |  |  |  |  |
| **Outcome: SBPV, standing** |  | |  |  |  |  |  |  |  |  |  |  |  |  |  |
| NPS | 0.009 [-0.014; 0.032] | | 0.446 |  | 0.011 [-0.013; 0.034] |  | 0.376 |  | 0.015 [-0.010; 0.039] |  | 0.236 |  | 0.014 [-0.010; 0.038] |  | 0.256 |
| BMI |  | |  |  | 0.013 [0.003; 0.022] |  | 0.012 |  |  |  |  |  | 0.014 [0.004; 0.024] |  | 0.007 |
| LTPA = 1 |  | |  |  | 0.031 [-0.114; 0.175] |  | 0.679 |  |  |  |  |  | 0.023 [-0.123; 0.169] |  | 0.761 |
| LTPA = 2 |  | |  |  | 0.139 [0.007; 0.271] |  | 0.040 |  |  |  |  |  | 0.133 [-0.000; 0.267] |  | 0.050 |
| LTPA = 3 |  | |  |  | 0.049 [-0.111; 0.208] |  | 0.549 |  |  |  |  |  | 0.039 [-0.122; 0.199] |  | 0.638 |
| Smoking = 1 |  | |  |  | -0.181 [-0.300; -0.061] |  | 0.003 |  |  |  |  |  | -0.175 [-0.295; -0.055] |  | 0.004 |
| Smoking = 2 |  | |  |  | -0.287 [-0.423; -0.151] |  | < 0.001 |  |  |  |  |  | -0.279 [-0.419; -0.142] |  | < 0.001 |
| HSCL-25 |  | |  |  |  |  |  |  | -0.117 [-0.274; 0.040] |  | 0.145 |  | -0.059 [-0.218; 0.099] |  | 0.463 |
| Comorbidity = 1 |  | |  |  |  |  |  |  | 0.021 [-0.147; 0.188] |  | 0.809 |  | 0.002 [-0.167; 0.171] |  | 0.982 |
| Medication = 1 |  | |  |  |  |  |  |  | -0.041 [-0.179; 0.098] |  | 0.564 |  | -0.075 [-0.215; 0.066] |  | 0.297 |
|  |  | |  |  |  |  |  |  |  |  |  |  |  |  |  |
| **Outcome: BRS, seated** |  | |  |  |  |  |  |  |  |  |  |  |  |  |  |
| NPS | -0.018 [-0.032; -0.004] | | 0.011 |  | -0.011 [-0.025; 0.002] |  | 0.108 |  | -0.010 [-0.024; 0.005] |  | 0.193 |  | -0.007 [-0.021; 0.007] |  | 0.344 |
| BMI |  | |  |  | -0.019 [-0.025; -0.014] |  | < 0.001 |  |  |  |  |  | -0.016 [-0.022; -0.010] |  | < 0.001 |
| LTPA = 1 |  | |  |  | 0.036 [-0.049; 0.122] |  | 0.408 |  |  |  |  |  | 0.018 [-0.068; 0.103] |  | 0.686 |
| LTPA = 2 |  | |  |  | -0.002 [-0.080; 0.076] |  | 0.961 |  |  |  |  |  | -0.011 [-0.089; 0.068] |  | 0.792 |
| LTPA = 3 |  | |  |  | 0.097 [0.003; 0.192] |  | 0.042 |  |  |  |  |  | 0.080 [-0.014; 0.174] |  | 0.097 |
| Smoking = 1 |  | |  |  | 0.082 [0.012; 0.153] |  | 0.022 |  |  |  |  |  | 0.087 [0.017; 0.157] |  | 0.015 |
| Smoking = 2 |  | |  |  | -0.059 [-0.139; 0.021] |  | 0.148 |  |  |  |  |  | -0.050 [-0.131; 0.030] |  | 0.221 |
| HSCL-25 |  | |  |  |  |  |  |  | -0.059 [-0.152; 0.034] |  | 0.214 |  | -0.036 [-0.129; 0.057] |  | 0.452 |
| Comorbidity = 1 |  | |  |  |  |  |  |  | -0.154 [-0.253; -0.055] |  | 0.002 |  | -0.095 [-0.194; 0.004] |  | 0.061 |
| Medication = 1 |  | |  |  |  |  |  |  | -0.179 [-0.261; -0.097] |  | < 0.001 |  | -0.132 [-0.214; -0.049] |  | 0.002 |
|  |  | |  |  |  |  |  |  |  |  |  |  |  |  |  |
| **Outcome: BRS, standing** |  | |  |  |  |  |  |  |  |  |  |  |  |  |  |
| NPS | -0.027 [-0.042; -0.012] | | < 0.001 |  | -0.019 [-0.033; -0.004] |  | 0.011 |  | -0.019 [-0.034; -0.003] |  | 0.017 |  | -0.015 [-0.030; 0.000] |  | 0.048 |
| BMI |  | |  |  | -0.026 [-0.032; -0.019] |  | < 0.001 |  |  |  |  |  | -0.023 [-0.029; -0.017] |  | < 0.001 |
| LTPA = 1 |  | |  |  | 0.013 [-0.077; 0.103] |  | 0.781 |  |  |  |  |  | 0.000 [-0.090; 0.090] |  | 0.998 |
| LTPA = 2 |  | |  |  | 0.013 [-0.069; 0.095] |  | 0.752 |  |  |  |  |  | 0.009 [-0.073; 0.092] |  | 0.828 |
| LTPA = 3 |  | |  |  | 0.182 [0.083; 0.281] |  | < 0.001 |  |  |  |  |  | 0.170 [0.070; 0.269] |  | 0.001 |
| Smoking = 1 |  | |  |  | 0.115 [0.041; 0.189] |  | 0.002 |  |  |  |  |  | 0.120 [0.046; 0.194] |  | 0.001 |
| Smoking = 2 |  | |  |  | 0.015 [-0.069; 0.100] |  | 0.723 |  |  |  |  |  | 0.021 [-0.064; 0.106] |  | 0.623 |
| HSCL-25 |  | |  |  |  |  |  |  | -0.058 [-0.158; 0.042] |  | 0.253 |  | -0.031 [-0.129; 0.067] |  | 0.539 |
| Comorbidity = 1 |  | |  |  |  |  |  |  | -0.094 [-0.201; 0.012] |  | 0.081 |  | -0.014 [-0.118; 0.091] |  | 0.797 |
| Medication = 1 |  | |  |  |  |  |  |  | -0.206 [-0.294; -0.118] |  | < 0.001 |  | -0.140 [-0.226; -0.053] |  | 0.002 |
