# Supplementary material for: Musculoskeletal pains and cardiovascular autonomic function in the general Northern Finnish population
Source: BMC Musculoskelet Disord. 2019 Jan 31;20:45. doi: 10.1186/s12891-019-2426-2 (PMC6357438; doi:10.1186/s12891-019-2426-2)
Supplement: Supplementary file 6 — Subanalysis 1, men. (DOCX 50 kb) [file 12891_2019_2426_MOESM6_ESM.docx]

**Additional file 6.** Subanalysis 1, men. Complete linear regression models for the association between number of pain sites (NPS) and cardiovascular autonomic function (HR, rMSSD, SBPV, BRS) among men in Subsample 1, i.e. men with intense (NRS ≥ 5) and frequent (> 30 days/year) pain (for HR and rMSSD, n = 450; for SBPV and BRS, n = 250). Variable coding, reference groups and model construction are presented in Additional files 1–3.

| Variables | Model I |  |  | Model II |  |  | Model III |  |  | Model IV |  |
| --- | --- | --- | --- | --- | --- | --- | --- | --- | --- | --- | --- |
|  | β [95% CI] | P |  | β [95% CI] | P |  | β [95% CI] | P |  | β [95% CI] | P |
| **Outcome: HR, seated** |  |  |  |  |  |  |  |  |  |  |  |
| NPS | -0.448 [-1.046; 0.149] | 0.141 |  | -0.782 [-1.371; -0.193] | 0.009 |  | -0.663 [-1.271; -0.056] | 0.032 |  | -0.902 [-1.501; -0.302] | 0.003 |
| BMI |  |  |  | 0.228 [-0.034; 0.489] | 0.088 |  |  |  |  | 0.229 [-0.037; 0.495] | 0.092 |
| LTPA = 1 |  |  |  | -3.559 [-6.617; -0.500] | 0.023 |  |  |  |  | -3.299 [-6.375; -0.222] | 0.036 |
| LTPA = 2 |  |  |  | -4.417 [-7.130; -1.704] | 0.001 |  |  |  |  | -4.078 [-6.812; -1.345] | 0.004 |
| LTPA = 3 |  |  |  | -7.636 [-11.192; -4.079] | < 0.001 |  |  |  |  | -7.342 [-10.928; -3.756] | < 0.001 |
| Smoking = 1 |  |  |  | -0.046 [-2.550; 2.458] | 0.971 |  |  |  |  | -0.257 [-2.770; 2.255] | 0.841 |
| Smoking = 2 |  |  |  | 3.735 [0.929; 6.540] | 0.009 |  |  |  |  | 3.168 [0.308; 6.029] | 0.030 |
| HSCL-25 |  |  |  |  |  |  | 5.557 [2.189; 8.925] | 0.001 |  | 3.579 [0.206; 6.953] | 0.038 |
| Comorbidity = 1 |  |  |  |  |  |  | 0.755 [-2.918; 4.428] | 0.686 |  | -0.193 [-3.834; 3.449] | 0.917 |
| Medication = 1 |  |  |  |  |  |  | 0.216 [-2.782; 3.214] | 0.888 |  | -0.457 [-3.403; 2.490] | 0.761 |
|  |  |  |  |  |  |  |  |  |  |  |  |
| **Outcome: HR, standing** |  |  |  |  |  |  |  |  |  |  |  |
| NPS | -0.349 [-1.017; 0.319] | 0.305 |  | -0.660 [-1.324; 0.003] | 0.051 |  | -0.643 [-1.320; 0.034] | 0.063 |  | -0.843 [-1.515; -0.171] | 0.014 |
| BMI |  |  |  | -0.056 [-0.350; 0.239] | 0.710 |  |  |  |  | -0.032 [-0.331; 0.266] | 0.831 |
| LTPA = 1 |  |  |  | -3.511 [-6.955; -0.068] | 0.046 |  |  |  |  | -3.096 [-6.545; 0.354] | 0.078 |
| LTPA = 2 |  |  |  | -4.503 [-7.557; -1.449] | 0.004 |  |  |  |  | -4.101 [-7.167; -1.036] | 0.009 |
| LTPA = 3 |  |  |  | -7.851 [-11.855; -3.848] | < 0.001 |  |  |  |  | -7.62 [-11.642; -3.599] | < 0.001 |
| Smoking = 1 |  |  |  | 0.108 [-2.711; 2.927] | 0.940 |  |  |  |  | -0.133 [-2.951; 2.685] | 0.926 |
| Smoking = 2 |  |  |  | 4.836 [1.678; 7.994] | 0.003 |  |  |  |  | 4.051 [0.843; 7.259] | 0.013 |
| HSCL-25 |  |  |  |  |  |  | 7.121 [3.369; 10.874] | < 0.001 |  | 4.989 [1.207; 8.772] | 0.010 |
| Comorbidity = 1 |  |  |  |  |  |  | -0.317 [-4.410; 3.776] | 0.879 |  | -0.833 [-4.916; 3.250] | 0.689 |
| Medication = 1 |  |  |  |  |  |  | -1.462 [-4.802; 1.878] | 0.390 |  | -1.819 [-5.123; 1.485] | 0.280 |
|  |  |  |  |  |  |  |  |  |  |  |  |
| **Outcome: rMSSD, seated** |  |  |  |  |  |  |  |  |  |  |  |
| NPS | 0.005 [-0.026; 0.037] | 0.747 |  | 0.022 [-0.009; 0.053] | 0.170 |  | 0.011 [-0.021; 0.044] | 0.495 |  | 0.024 [-0.008; 0.056] | 0.145 |
| BMI |  |  |  | -0.021 [-0.035; -0.008] | 0.003 |  |  |  |  | -0.022 [-0.036; -0.007] | 0.003 |
| LTPA = 1 |  |  |  | 0.138 [-0.024; 0.301] | 0.095 |  |  |  |  | 0.127 [-0.037; 0.290] | 0.130 |
| LTPA = 2 |  |  |  | 0.212 [0.068; 0.356] | 0.004 |  |  |  |  | 0.202 [0.056; 0.347] | 0.007 |
| LTPA = 3 |  |  |  | 0.329 [0.140; 0.518] | 0.001 |  |  |  |  | 0.314 [0.123; 0.505] | 0.001 |
| Smoking = 1 |  |  |  | -0.079 [-0.212; 0.054] | 0.242 |  |  |  |  | -0.075 [-0.209; 0.058] | 0.268 |
| Smoking = 2 |  |  |  | -0.196 [-0.345; -0.047] | 0.010 |  |  |  |  | -0.191 [-0.343; -0.039] | 0.014 |
| HSCL-25 |  |  |  |  |  |  | -0.182 [-0.361; -0.002] | 0.047 |  | -0.077 [-0.256; 0.103] | 0.402 |
| Comorbidity = 1 |  |  |  |  |  |  | 0.011 [-0.184; 0.207] | 0.909 |  | 0.088 [-0.105; 0.282] | 0.370 |
| Medication = 1 |  |  |  |  |  |  | -0.118 [-0.278; 0.041] | 0.146 |  | -0.073 [-0.230; 0.083] | 0.358 |
|  |  |  |  |  |  |  |  |  |  |  |  |
| **Outcome: rMSSD, standing** |  |  |  |  |  |  |  |  |  |  |  |
| NPS | 0.005 [-0.026; 0.037] | 0.748 |  | 0.017 [-0.014; 0.049] | 0.277 |  | 0.013 [-0.019; 0.045] | 0.436 |  | 0.021 [-0.011; 0.053] | 0.205 |
| BMI |  |  |  | -0.011 [-0.025; 0.003] | 0.137 |  |  |  |  | -0.01 [-0.024; 0.004] | 0.166 |
| LTPA = 1 |  |  |  | 0.045 [-0.119; 0.209] | 0.590 |  |  |  |  | 0.032 [-0.133; 0.197] | 0.701 |
| LTPA = 2 |  |  |  | 0.101 [-0.044; 0.247] | 0.171 |  |  |  |  | 0.084 [-0.062; 0.231] | 0.260 |
| LTPA = 3 |  |  |  | 0.267 [0.076; 0.457] | 0.006 |  |  |  |  | 0.243 [0.051; 0.435] | 0.013 |
| Smoking = 1 |  |  |  | -0.063 [-0.197; 0.071] | 0.356 |  |  |  |  | -0.054 [-0.189; 0.081] | 0.429 |
| Smoking = 2 |  |  |  | -0.247 [-0.397; -0.097] | 0.001 |  |  |  |  | -0.232 [-0.385; -0.078] | 0.003 |
| HSCL-25 |  |  |  |  |  |  | -0.220 [-0.398; -0.042] | 0.016 |  | -0.132 [-0.313; 0.049] | 0.154 |
| Comorbidity = 1 |  |  |  |  |  |  | 0.002 [-0.192; 0.196] | 0.984 |  | 0.059 [-0.137; 0.254] | 0.556 |
| Medication = 1 |  |  |  |  |  |  | -0.113 [-0.272; 0.046] | 0.163 |  | -0.090 [-0.248; 0.068] | 0.263 |
|  |  |  |  |  |  |  |  |  |  |  |  |
| **Outcome: SBPV, seated** |  |  |  |  |  |  |  |  |  |  |  |
| NPS | -0.013 [-0.066; 0.039] | 0.613 |  | -0.010 [-0.063; 0.043] | 0.715 |  | -0.009 [-0.063; 0.046] | 0.754 |  | -0.010 [-0.064; 0.045] | 0.733 |
| BMI |  |  |  | 0.012 [-0.012; 0.036] | 0.311 |  |  |  |  | 0.013 [-0.011; 0.037] | 0.295 |
| LTPA = 1 |  |  |  | 0.095 [-0.191; 0.381] | 0.514 |  |  |  |  | 0.093 [-0.195; 0.381] | 0.525 |
| LTPA = 2 |  |  |  | 0.094 [-0.151; 0.338] | 0.450 |  |  |  |  | 0.077 [-0.171; 0.326] | 0.541 |
| LTPA = 3 |  |  |  | -0.21 [-0.534; 0.115] | 0.204 |  |  |  |  | -0.23 [-0.559; 0.099] | 0.170 |
| Smoking = 1 |  |  |  | 0.081 [-0.142; 0.305] | 0.473 |  |  |  |  | 0.086 [-0.140; 0.312] | 0.453 |
| Smoking = 2 |  |  |  | -0.198 [-0.458; 0.062] | 0.135 |  |  |  |  | -0.203 [-0.476; 0.070] | 0.144 |
| HSCL-25 |  |  |  |  |  |  | -0.115 [-0.401; 0.172] | 0.432 |  | -0.035 [-0.333; 0.263] | 0.818 |
| Comorbidity = 1 |  |  |  |  |  |  | 0.117 [-0.281; 0.514] | 0.564 |  | 0.090 [-0.307; 0.487] | 0.654 |
| Medication = 1 |  |  |  |  |  |  | -0.058 [-0.326; 0.209] | 0.667 |  | -0.129 [-0.401; 0.143] | 0.351 |
|  |  |  |  |  |  |  |  |  |  |  |  |
| **Outcome: SBPV, standing** |  |  |  |  |  |  |  |  |  |  |  |
| NPS | -0.042 [-0.094; 0.009] | 0.109 |  | -0.030 [-0.083; 0.022] | 0.258 |  | -0.029 [-0.081; 0.024] | 0.283 |  | -0.022 [-0.075; 0.030] | 0.404 |
| BMI |  |  |  | 0.008 [-0.016; 0.031] | 0.527 |  |  |  |  | 0.011 [-0.012; 0.035] | 0.348 |
| LTPA = 1 |  |  |  | 0.283 [0.000; 0.565] | 0.050 |  |  |  |  | 0.281 [0.003; 0.558] | 0.047 |
| LTPA = 2 |  |  |  | 0.161 [-0.080; 0.402] | 0.189 |  |  |  |  | 0.096 [-0.143; 0.336] | 0.429 |
| LTPA = 3 |  |  |  | -0.039 [-0.359; 0.281] | 0.810 |  |  |  |  | -0.102 [-0.419; 0.216] | 0.529 |
| Smoking = 1 |  |  |  | 0.104 [-0.116; 0.324] | 0.353 |  |  |  |  | 0.141 [-0.077; 0.359] | 0.205 |
| Smoking = 2 |  |  |  | -0.181 [-0.437; 0.075] | 0.166 |  |  |  |  | -0.139 [-0.402; 0.124] | 0.300 |
| HSCL-25 |  |  |  |  |  |  | -0.356 [-0.634; -0.078] | 0.012 |  | -0.295 [-0.582; -0.007] | 0.045 |
| Comorbidity = 1 |  |  |  |  |  |  | 0.088 [-0.297; 0.473] | 0.653 |  | 0.053 [-0.330; 0.436] | 0.786 |
| Medication = 1 |  |  |  |  |  |  | -0.271 [-0.530; -0.013] | 0.040 |  | -0.337 [-0.600; -0.075] | 0.012 |
|  |  |  |  |  |  |  |  |  |  |  |  |
| **Outcome: BRS, seated** |  |  |  |  |  |  |  |  |  |  |  |
| NPS | 0.018 [-0.017; 0.053] | 0.319 |  | 0.035 [0.001; 0.068] | 0.043 |  | 0.020 [-0.016; 0.056] | 0.275 |  | 0.034 [-0.001; 0.068] | 0.057 |
| BMI |  |  |  | -0.028 [-0.043; -0.013] | < 0.001 |  |  |  |  | -0.028 [-0.043; -0.012] | 0.001 |
| LTPA = 1 |  |  |  | 0.143 [-0.038; 0.324] | 0.121 |  |  |  |  | 0.146 [-0.036; 0.328] | 0.116 |
| LTPA = 2 |  |  |  | 0.114 [-0.041; 0.268] | 0.148 |  |  |  |  | 0.114 [-0.043; 0.271] | 0.155 |
| LTPA = 3 |  |  |  | 0.311 [0.106; 0.516] | 0.003 |  |  |  |  | 0.309 [0.100; 0.517] | 0.004 |
| Smoking = 1 |  |  |  | -0.170 [-0.311; -0.029] | 0.019 |  |  |  |  | -0.169 [-0.312; -0.026] | 0.021 |
| Smoking = 2 |  |  |  | -0.211 [-0.376; -0.047] | 0.012 |  |  |  |  | -0.213 [-0.386; -0.041] | 0.016 |
| HSCL-25 |  |  |  |  |  |  | -0.068 [-0.258; 0.123] | 0.486 |  | 0.023 [-0.166; 0.211] | 0.814 |
| Comorbidity = 1 |  |  |  |  |  |  | -0.160 [-0.425; 0.105] | 0.235 |  | -0.091 [-0.342; 0.161] | 0.478 |
| Medication = 1 |  |  |  |  |  |  | -0.039 [-0.217; 0.139] | 0.664 |  | 0.008 [-0.164; 0.180] | 0.925 |
|  |  |  |  |  |  |  |  |  |  |  |  |
| **Outcome: BRS, standing** |  |  |  |  |  |  |  |  |  |  |  |
| NPS | 0.016 [-0.021; 0.053] | 0.391 |  | 0.030 [-0.007; 0.066] | 0.111 |  | 0.020 [-0.018; 0.058] | 0.301 |  | 0.029 [-0.008; 0.067] | 0.126 |
| BMI |  |  |  | -0.019 [-0.036; -0.003] | 0.024 |  |  |  |  | -0.019 [-0.036; -0.002] | 0.028 |
| LTPA = 1 |  |  |  | 0.052 [-0.144; 0.248] | 0.603 |  |  |  |  | 0.052 [-0.146; 0.249] | 0.607 |
| LTPA = 2 |  |  |  | 0.066 [-0.101; 0.234] | 0.437 |  |  |  |  | 0.061 [-0.110; 0.232] | 0.483 |
| LTPA = 3 |  |  |  | 0.238 [0.015; 0.460] | 0.036 |  |  |  |  | 0.230 [0.004; 0.456] | 0.046 |
| Smoking = 1 |  |  |  | -0.152 [-0.305; 0.001] | 0.052 |  |  |  |  | -0.151 [-0.306; 0.004] | 0.057 |
| Smoking = 2 |  |  |  | -0.276 [-0.454; -0.098] | 0.003 |  |  |  |  | -0.280 [-0.467; -0.092] | 0.004 |
| HSCL-25 |  |  |  |  |  |  | -0.104 [-0.305; 0.098] | 0.312 |  | -0.002 [-0.207; 0.203] | 0.983 |
| Comorbidity = 1 |  |  |  |  |  |  | -0.036 [-0.316; 0.244] | 0.800 |  | 0.023 [-0.250; 0.296] | 0.867 |
| Medication = 1 |  |  |  |  |  |  | -0.069 [-0.257; 0.119] | 0.469 |  | -0.047 [-0.234; 0.140] | 0.624 |
