# Supplementary material for: Musculoskeletal pains and cardiovascular autonomic function in the general Northern Finnish population
Source: BMC Musculoskelet Disord. 2019 Jan 31;20:45. doi: 10.1186/s12891-019-2426-2 (PMC6357438; doi:10.1186/s12891-019-2426-2)
Supplement: Supplementary file 7 — Subanalysis 1, women. (DOCX 50 kb) [file 12891_2019_2426_MOESM7_ESM.docx]

**Additional file 7.** Subanalysis 1, women. Complete linear regression models for the association between number of pain sites (NPS) and cardiovascular autonomic function (HR, rMSSD, SBPV, BRS) among women in Subsample 1, i.e. women with intense (NRS ≥ 5) and frequent (> 30 days/year) pain (for HR and rMSSD, n = 761; for SBPV and BRS, n = 375). Variable coding, reference groups and model construction are presented in Additional files 1–3.

| Variables | Model I |  |  | Model II |  |  | Model III |  |  | Model IV |  |
| --- | --- | --- | --- | --- | --- | --- | --- | --- | --- | --- | --- |
|  | β [95% CI] | P |  | β [95% CI] | P |  | β [95% CI] | P |  | β [95% CI] | P |
| **Outcome: HR, seated** |  |  |  |  |  |  |  |  |  |  |  |
| NPS | 0.497 [0.108; 0.886] | 0.012 |  | 0.300 [-0.083; 0.683] | 0.125 |  | 0.370 [-0.027; 0.767] | 0.068 |  | 0.239 [-0.152; 0.629] | 0.231 |
| BMI |  |  |  | 0.357 [0.214; 0.499] | < 0.001 |  |  |  |  | 0.303 [0.155; 0.452] | < 0.001 |
| LTPA = 1 |  |  |  | 1.184 [-0.885; 3.253] | 0.262 |  |  |  |  | 1.349 [-0.721; 3.419] | 0.201 |
| LTPA = 2 |  |  |  | -0.362 [-2.221; 1.496] | 0.702 |  |  |  |  | -0.341 [-2.202; 1.519] | 0.719 |
| LTPA = 3 |  |  |  | -2.869 [-5.234; -0.505] | 0.017 |  |  |  |  | -2.685 [-5.051; -0.319] | 0.026 |
| Smoking = 1 |  |  |  | -3.096 [-4.747; -1.445] | < 0.001 |  |  |  |  | -3.038 [-4.687; -1.388] | < 0.001 |
| Smoking = 2 |  |  |  | -1.855 [-3.750; 0.041] | 0.055 |  |  |  |  | -1.741 [-3.643; 0.161] | 0.073 |
| HSCL-25 |  |  |  |  |  |  | -0.203 [-2.180; 1.775] | 0.841 |  | -0.280 [-2.24; 1.681] | 0.779 |
| Comorbidity = 1 |  |  |  |  |  |  | 3.930 [1.685; 6.176] | 0.001 |  | 2.936 [0.711; 5.16] | 0.010 |
| Medication = 1 |  |  |  |  |  |  | 1.873 [-0.039; 3.784] | 0.055 |  | 1.022 [-0.900; 2.944] | 0.297 |
|  |  |  |  |  |  |  |  |  |  |  |  |
| **Outcome: HR, standing** |  |  |  |  |  |  |  |  |  |  |  |
| NPS | 0.525 [0.058; 0.992] | 0.028 |  | 0.402 [-0.066; 0.869] | 0.092 |  | 0.482 [0.002; 0.963] | 0.049 |  | 0.396 [-0.083; 0.874] | 0.105 |
| BMI |  |  |  | 0.227 [0.053; 0.401] | 0.011 |  |  |  |  | 0.196 [0.014; 0.377] | 0.034 |
| LTPA = 1 |  |  |  | 1.356 [-1.168; 3.881] | 0.292 |  |  |  |  | 1.460 [-1.075; 3.996] | 0.258 |
| LTPA = 2 |  |  |  | -0.300 [-2.567; 1.968] | 0.795 |  |  |  |  | -0.376 [-2.655; 1.903] | 0.746 |
| LTPA = 3 |  |  |  | -2.627 [-5.512; 0.258] | 0.074 |  |  |  |  | -2.594 [-5.492; 0.304] | 0.079 |
| Smoking = 1 |  |  |  | -3.224 [-5.238; -1.210] | 0.002 |  |  |  |  | -3.128 [-5.149; -1.107] | 0.002 |
| Smoking = 2 |  |  |  | -3.418 [-5.731; -1.105] | 0.004 |  |  |  |  | -3.251 [-5.581; -0.922] | 0.006 |
| HSCL-25 |  |  |  |  |  |  | -1.249 [-3.643; 1.144] | 0.306 |  | -1.123 [-3.525; 1.278] | 0.359 |
| Comorbidity = 1 |  |  |  |  |  |  | 2.665 [-0.052; 5.383] | 0.055 |  | 1.854 [-0.870; 4.578] | 0.182 |
| Medication = 1 |  |  |  |  |  |  | 1.059 [-1.255; 3.372] | 0.369 |  | 0.597 [-1.757; 2.951] | 0.618 |
|  |  |  |  |  |  |  |  |  |  |  |  |
| **Outcome: rMSSD, seated** |  |  |  |  |  |  |  |  |  |  |  |
| NPS | -0.032 [-0.055; -0.009] | 0.007 |  | -0.019 [-0.042; 0.004] | 0.106 |  | -0.018 [-0.042; 0.005] | 0.126 |  | -0.011 [-0.034; 0.012] | 0.362 |
| BMI |  |  |  | -0.025 [-0.033; -0.016] | < 0.001 |  |  |  |  | -0.020 [-0.029; -0.011] | < 0.001 |
| LTPA = 1 |  |  |  | -0.041 [-0.165; 0.083] | 0.520 |  |  |  |  | -0.058 [-0.181; 0.066] | 0.359 |
| LTPA = 2 |  |  |  | 0.050 [-0.062; 0.161] | 0.380 |  |  |  |  | 0.037 [-0.074; 0.148] | 0.511 |
| LTPA = 3 |  |  |  | 0.151 [0.009; 0.293] | 0.037 |  |  |  |  | 0.125 [-0.016; 0.266] | 0.082 |
| Smoking = 1 |  |  |  | 0.113 [0.014; 0.212] | 0.026 |  |  |  |  | 0.115 [0.017; 0.213] | 0.022 |
| Smoking = 2 |  |  |  | 0.058 [-0.056; 0.171] | 0.321 |  |  |  |  | 0.062 [-0.051; 0.175] | 0.284 |
| HSCL-25 |  |  |  |  |  |  | -0.097 [-0.215; 0.020] | 0.103 |  | -0.086 [-0.202; 0.031] | 0.151 |
| Comorbidity = 1 |  |  |  |  |  |  | -0.263 [-0.396; -0.130] | < 0.001 |  | -0.205 [-0.337; -0.072] | 0.002 |
| Medication = 1 |  |  |  |  |  |  | -0.189 [-0.302; -0.076] | 0.001 |  | -0.132 [-0.247; -0.018] | 0.024 |
|  |  |  |  |  |  |  |  |  |  |  |  |
| **Outcome: rMSSD, standing** |  |  |  |  |  |  |  |  |  |  |  |
| NPS | -0.022 [-0.046; 0.002] | 0.075 |  | -0.013 [-0.037; 0.011] | 0.291 |  | -0.011 [-0.035; 0.014] | 0.389 |  | -0.006 [-0.031; 0.018] | 0.617 |
| BMI |  |  |  | -0.017 [-0.026; -0.008] | < 0.001 |  |  |  |  | -0.012 [-0.021; -0.002] | 0.013 |
| LTPA = 1 |  |  |  | -0.048 [-0.178; 0.082] | 0.466 |  |  |  |  | -0.065 [-0.194; 0.064] | 0.325 |
| LTPA = 2 |  |  |  | 0.041 [-0.076; 0.158] | 0.490 |  |  |  |  | 0.033 [-0.083; 0.150] | 0.574 |
| LTPA = 3 |  |  |  | 0.138 [-0.011; 0.286] | 0.069 |  |  |  |  | 0.116 [-0.032; 0.264] | 0.125 |
| Smoking = 1 |  |  |  | 0.058 [-0.046; 0.162] | 0.272 |  |  |  |  | 0.057 [-0.046; 0.160] | 0.278 |
| Smoking = 2 |  |  |  | 0.046 [-0.073; 0.165] | 0.444 |  |  |  |  | 0.044 [-0.074; 0.163] | 0.464 |
| HSCL-25 |  |  |  |  |  |  | -0.045 [-0.166; 0.077] | 0.469 |  | -0.032 [-0.154; 0.091] | 0.609 |
| Comorbidity = 1 |  |  |  |  |  |  | -0.262 [-0.400; -0.125] | < 0.001 |  | -0.225 [-0.364; -0.086] | 0.002 |
| Medication = 1 |  |  |  |  |  |  | -0.155 [-0.272; -0.038] | 0.010 |  | -0.122 [-0.242; -0.002] | 0.046 |
|  |  |  |  |  |  |  |  |  |  |  |  |
| **Outcome: SBPV, seated** |  |  |  |  |  |  |  |  |  |  |  |
| NPS | 0.023 [-0.02; 0.066] | 0.301 |  | 0.030 [-0.014; 0.073] | 0.182 |  | 0.039 [-0.006; 0.084] | 0.086 |  | 0.041 [-0.004; 0.086] | 0.071 |
| BMI |  |  |  | -0.013 [-0.029; 0.003] | 0.101 |  |  |  |  | -0.007 [-0.024; 0.009] | 0.377 |
| LTPA = 1 |  |  |  | 0.084 [-0.149; 0.317] | 0.479 |  |  |  |  | 0.054 [-0.181; 0.288] | 0.653 |
| LTPA = 2 |  |  |  | 0.131 [-0.075; 0.337] | 0.213 |  |  |  |  | 0.106 [-0.101; 0.312] | 0.316 |
| LTPA = 3 |  |  |  | -0.028 [-0.291; 0.235] | 0.837 |  |  |  |  | -0.080 [-0.345; 0.185] | 0.552 |
| Smoking = 1 |  |  |  | 0.097 [-0.093; 0.287] | 0.316 |  |  |  |  | 0.105 [-0.086; 0.295] | 0.280 |
| Smoking = 2 |  |  |  | -0.042 [-0.253; 0.169] | 0.695 |  |  |  |  | -0.027 [-0.240; 0.187] | 0.805 |
| HSCL-25 |  |  |  |  |  |  | -0.155 [-0.382; 0.072] | 0.179 |  | -0.135 [-0.369; 0.099] | 0.257 |
| Comorbidity = 1 |  |  |  |  |  |  | -0.271 [-0.507; -0.036] | 0.024 |  | -0.254 [-0.497; -0.011] | 0.040 |
| Medication = 1 |  |  |  |  |  |  | -0.103 [-0.300; 0.094] | 0.306 |  | -0.091 [-0.295; 0.114] | 0.385 |
|  |  |  |  |  |  |  |  |  |  |  |  |
| **Outcome: SBPV, standing** |  |  |  |  |  |  |  |  |  |  |  |
| NPS | 0.029 [-0.014; 0.072] | 0.186 |  | 0.032 [-0.011; 0.076] | 0.146 |  | 0.040 [-0.005; 0.086] | 0.078 |  | 0.038 [-0.007; 0.084] | 0.097 |
| BMI |  |  |  | -0.005 [-0.020; 0.011] | 0.561 |  |  |  |  | -0.002 [-0.019; 0.014] | 0.769 |
| LTPA = 1 |  |  |  | 0.072 [-0.162; 0.305] | 0.546 |  |  |  |  | 0.063 [-0.173; 0.300] | 0.598 |
| LTPA = 2 |  |  |  | 0.095 [-0.111; 0.302] | 0.365 |  |  |  |  | 0.083 [-0.125; 0.292] | 0.433 |
| LTPA = 3 |  |  |  | 0.156 [-0.108; 0.420] | 0.246 |  |  |  |  | 0.131 [-0.136; 0.399] | 0.335 |
| Smoking = 1 |  |  |  | -0.103 [-0.293; 0.088] | 0.290 |  |  |  |  | -0.103 [-0.295; 0.089] | 0.293 |
| Smoking = 2 |  |  |  | -0.253 [-0.465; -0.041] | 0.019 |  |  |  |  | -0.249 [-0.465; -0.034] | 0.024 |
| HSCL-25 |  |  |  |  |  |  | -0.141 [-0.370; 0.089] | 0.229 |  | -0.068 [-0.304; 0.168] | 0.570 |
| Comorbidity = 1 |  |  |  |  |  |  | -0.152 [-0.390; 0.086] | 0.210 |  | -0.141 [-0.386; 0.104] | 0.259 |
| Medication = 1 |  |  |  |  |  |  | -0.025 [-0.224; 0.175] | 0.808 |  | 0.005 [-0.201; 0.212] | 0.961 |
|  |  |  |  |  |  |  |  |  |  |  |  |
| **Outcome: BRS, seated** |  |  |  |  |  |  |  |  |  |  |  |
| NPS | -0.033 [-0.06; -0.007] | 0.014 |  | -0.024 [-0.050; 0.002] | 0.071 |  | -0.023 [-0.050; 0.004] | 0.095 |  | -0.019 [-0.046; 0.008] | 0.167 |
| BMI |  |  |  | -0.016 [-0.025; -0.006] | 0.001 |  |  |  |  | -0.011 [-0.020; -0.001] | 0.035 |
| LTPA = 1 |  |  |  | -0.021 [-0.161; 0.120] | 0.773 |  |  |  |  | -0.056 [-0.195; 0.084] | 0.431 |
| LTPA = 2 |  |  |  | -0.028 [-0.151; 0.096] | 0.662 |  |  |  |  | -0.041 [-0.164; 0.082] | 0.514 |
| LTPA = 3 |  |  |  | 0.076 [-0.082; 0.234] | 0.343 |  |  |  |  | 0.044 [-0.114; 0.202] | 0.585 |
| Smoking = 1 |  |  |  | 0.105 [-0.010; 0.219] | 0.072 |  |  |  |  | 0.119 [0.006; 0.233] | 0.040 |
| Smoking = 2 |  |  |  | -0.100 [-0.227; 0.027] | 0.123 |  |  |  |  | -0.083 [-0.210; 0.044] | 0.200 |
| HSCL-25 |  |  |  |  |  |  | -0.074 [-0.211; 0.063] | 0.289 |  | -0.050 [-0.189; 0.090] | 0.484 |
| Comorbidity = 1 |  |  |  |  |  |  | -0.147 [-0.289; -0.005] | 0.043 |  | -0.106 [-0.250; 0.039] | 0.152 |
| Medication = 1 |  |  |  |  |  |  | -0.213 [-0.332; -0.094] | < 0.001 |  | -0.195 [-0.317; -0.073] | 0.002 |
|  |  |  |  |  |  |  |  |  |  |  |  |
| **Outcome: BRS, standing** |  |  |  |  |  |  |  |  |  |  |  |
| NPS | -0.038 [-0.066; -0.010] | 0.007 |  | -0.026 [-0.053; 0.002] | 0.067 |  | -0.030 [-0.058; -0.001] | 0.040 |  | -0.023 [-0.050; 0.005] | 0.111 |
| BMI |  |  |  | -0.024 [-0.034; -0.014] | < 0.001 |  |  |  |  | -0.019 [-0.029; -0.008] | < 0.001 |
| LTPA = 1 |  |  |  | -0.065 [-0.211; 0.080] | 0.379 |  |  |  |  | -0.103 [-0.248; 0.042] | 0.164 |
| LTPA = 2 |  |  |  | 0.008 [-0.121; 0.136] | 0.908 |  |  |  |  | -0.001 [-0.129; 0.127] | 0.991 |
| LTPA = 3 |  |  |  | 0.102 [-0.062; 0.267] | 0.223 |  |  |  |  | 0.075 [-0.089; 0.240] | 0.367 |
| Smoking = 1 |  |  |  | 0.123 [0.004; 0.242] | 0.043 |  |  |  |  | 0.137 [0.019; 0.255] | 0.023 |
| Smoking = 2 |  |  |  | 0.020 [-0.112; 0.152] | 0.763 |  |  |  |  | 0.033 [-0.099; 0.165] | 0.621 |
| HSCL-25 |  |  |  |  |  |  | -0.023 [-0.167; 0.121] | 0.758 |  | -0.003 [-0.147; 0.142] | 0.971 |
| Comorbidity = 1 |  |  |  |  |  |  | -0.162 [-0.311; -0.012] | 0.034 |  | -0.088 [-0.238; 0.063] | 0.252 |
| Medication = 1 |  |  |  |  |  |  | -0.256 [-0.381; -0.131] | < 0.001 |  | -0.229 [-0.356; -0.103] | < 0.001 |
