# Supplementary material for: Musculoskeletal pains and cardiovascular autonomic function in the general Northern Finnish population
Source: BMC Musculoskelet Disord. 2019 Jan 31;20:45. doi: 10.1186/s12891-019-2426-2 (PMC6357438; doi:10.1186/s12891-019-2426-2)
Supplement: Supplementary file 8 — Subanalysis 2, men. (DOCX 48 kb) [file 12891_2019_2426_MOESM8_ESM.docx]

**Additional file 8.** Subanalysis 2, men. Complete linear regression models for the association between number of pain sites (NPS) and cardiovascular autonomic function (HR, rMSSD, SBPV, BRS) among men in Subsample 2, i.e. men with clinically relevant symptoms of depression and anxiety (HSCL-25 score ≥ 1.55) (for HR and rMSSD, n = 302; for SBPV and BRS, n = 163). Variable coding, reference groups and model construction are presented in Additional files 1–3.

| Variables | Model I |  |  | Model II |  |  | Model III |  |  | Model IV |  |
| --- | --- | --- | --- | --- | --- | --- | --- | --- | --- | --- | --- |
|  | β [95% CI] | P |  | β [95% CI] | P |  | β [95% CI] | P |  | β [95% CI] | P |
| **Outcome: HR, seated** |  |  |  |  |  |  |  |  |  |  |  |
| NPS | 0.040 [-0.669; 0.748] | 0.912 |  | -0.209 [-0.900; 0.482] | 0.552 |  | 0.131 [-0.579; 0.840] | 0.717 |  | -0.141 [-0.840; 0.559] | 0.692 |
| BMI |  |  |  | 0.498 [0.202; 0.794] | 0.001 |  |  |  |  | 0.454 [0.135; 0.772] | 0.005 |
| LTPA = 1 |  |  |  | -3.004 [-6.931; 0.924] | 0.133 |  |  |  |  | -3.236 [-7.180; 0.707] | 0.107 |
| LTPA = 2 |  |  |  | -4.074 [-7.576; -0.573] | 0.023 |  |  |  |  | -3.969 [-7.487; -0.452] | 0.027 |
| LTPA = 3 |  |  |  | -8.937 [-14.188; -3.687] | 0.001 |  |  |  |  | -8.925 [-14.178; -3.673] | 0.001 |
| Smoking = 1 |  |  |  | -0.150 [-3.666; 3.366] | 0.933 |  |  |  |  | -0.242 [-3.771; 3.286] | 0.892 |
| Smoking = 2 |  |  |  | 3.950 [0.282; 7.617] | 0.035 |  |  |  |  | 3.908 [0.235; 7.582] | 0.037 |
| Comorbidity = 1 |  |  |  |  |  |  | 4.898 [0.578; 9.218] | 0.026 |  | 2.861 [-1.512; 7.233] | 0.199 |
| Medication = 1 |  |  |  |  |  |  | 0.255 [-3.530; 4.041] | 0.894 |  | -1.117 [-4.826; 2.592] | 0.554 |
|  |  |  |  |  |  |  |  |  |  |  |  |
| **Outcome: HR, standing** |  |  |  |  |  |  |  |  |  |  |  |
| NPS | 0.020 [-0.768; 0.807] | 0.961 |  | -0.180 [-0.963; 0.603] | 0.652 |  | 0.080 [-0.714; 0.873] | 0.843 |  | -0.140 [-0.934; 0.653] | 0.728 |
| BMI |  |  |  | 0.322 [-0.014; 0.658] | 0.060 |  |  |  |  | 0.318 [-0.044; 0.679] | 0.085 |
| LTPA = 1 |  |  |  | -3.441 [-7.891; 1.009] | 0.129 |  |  |  |  | -3.607 [-8.081; 0.867] | 0.114 |
| LTPA = 2 |  |  |  | -3.373 [-7.341; 0.594] | 0.095 |  |  |  |  | -3.379 [-7.370; 0.612] | 0.097 |
| LTPA = 3 |  |  |  | -8.682 [-14.631; -2.733] | 0.004 |  |  |  |  | -8.691 [-14.65; -2.732] | 0.004 |
| Smoking = 1 |  |  |  | -1.091 [-5.075; 2.893] | 0.590 |  |  |  |  | -1.087 [-5.090; 2.916] | 0.594 |
| Smoking = 2 |  |  |  | 4.097 [-0.059; 8.252] | 0.053 |  |  |  |  | 4.117 [-0.050; 8.285] | 0.053 |
| Comorbidity = 1 |  |  |  |  |  |  | 3.062 [-1.768; 7.893] | 0.213 |  | 1.778 [-3.182; 6.739] | 0.481 |
| Medication = 1 |  |  |  |  |  |  | -0.843 [-5.076; 3.390] | 0.695 |  | -1.838 [-6.046; 2.370] | 0.391 |
|  |  |  |  |  |  |  |  |  |  |  |  |
| **Outcome: rMSSD, seated** |  |  |  |  |  |  |  |  |  |  |  |
| NPS | 0.010 [-0.027; 0.047] | 0.596 |  | 0.026 [-0.011; 0.062] | 0.165 |  | 0.006 [-0.031; 0.044] | 0.738 |  | 0.024 [-0.013; 0.061] | 0.21 |
| BMI |  |  |  | -0.025 [-0.04; -0.009] | 0.002 |  |  |  |  | -0.023 [-0.04; -0.006] | 0.008 |
| LTPA = 1 |  |  |  | 0.123 [-0.084; 0.330] | 0.243 |  |  |  |  | 0.13 [-0.078; 0.339] | 0.221 |
| LTPA = 2 |  |  |  | 0.268 [0.083; 0.452] | 0.005 |  |  |  |  | 0.263 [0.077; 0.449] | 0.006 |
| LTPA = 3 |  |  |  | 0.357 [0.080; 0.634] | 0.012 |  |  |  |  | 0.356 [0.078; 0.634] | 0.012 |
| Smoking = 1 |  |  |  | -0.078 [-0.264; 0.107] | 0.408 |  |  |  |  | -0.074 [-0.26; 0.113] | 0.436 |
| Smoking = 2 |  |  |  | -0.260 [-0.453; -0.066] | 0.009 |  |  |  |  | -0.257 [-0.452; -0.063] | 0.01 |
| Comorbidity = 1 |  |  |  |  |  |  | -0.206 [-0.434; 0.021] | 0.075 |  | -0.09 [-0.321; 0.141] | 0.443 |
| Medication = 1 |  |  |  |  |  |  | -0.062 [-0.262; 0.137] | 0.538 |  | 0.012 [-0.184; 0.209] | 0.901 |
|  |  |  |  |  |  |  |  |  |  |  |  |
| **Outcome: rMSSD, standing** |  |  |  |  |  |  |  |  |  |  |  |
| NPS | 0.000 [-0.036; 0.037] | 0.981 |  | 0.012 [-0.024; 0.049] | 0.511 |  | -0.002 [-0.039; 0.035] | 0.905 |  | 0.010 [-0.028; 0.047] | 0.608 |
| BMI |  |  |  | -0.019 [-0.035; -0.004] | 0.017 |  |  |  |  | -0.015 [-0.032; 0.002] | 0.081 |
| LTPA = 1 |  |  |  | 0.095 [-0.114; 0.305] | 0.370 |  |  |  |  | 0.101 [-0.109; 0.310] | 0.347 |
| LTPA = 2 |  |  |  | 0.214 [0.027; 0.400] | 0.025 |  |  |  |  | 0.202 [0.015; 0.389] | 0.035 |
| LTPA = 3 |  |  |  | 0.250 [-0.029; 0.530] | 0.079 |  |  |  |  | 0.248 [-0.032; 0.527] | 0.082 |
| Smoking = 1 |  |  |  | -0.023 [-0.210; 0.164] | 0.808 |  |  |  |  | -0.013 [-0.201; 0.175] | 0.893 |
| Smoking = 2 |  |  |  | -0.217 [-0.412; -0.022] | 0.030 |  |  |  |  | -0.210 [-0.406; -0.015] | 0.035 |
| Comorbidity = 1 |  |  |  |  |  |  | -0.169 [-0.393; 0.056] | 0.141 |  | -0.094 [-0.327; 0.139] | 0.426 |
| Medication = 1 |  |  |  |  |  |  | -0.147 [-0.344; 0.050] | 0.143 |  | -0.098 [-0.295; 0.100] | 0.332 |
|  |  |  |  |  |  |  |  |  |  |  |  |
| **Outcome: SBPV, seated** |  |  |  |  |  |  |  |  |  |  |  |
| NPS | -0.011 [-0.072; 0.049] | 0.719 |  | -0.008 [-0.071; 0.054] | 0.792 |  | 0.001 [-0.061; 0.062] | 0.982 |  | 0.004 [-0.060; 0.068] | 0.910 |
| BMI |  |  |  | -0.008 [-0.036; 0.021] | 0.584 |  |  |  |  | -0.009 [-0.039; 0.021] | 0.549 |
| LTPA = 1 |  |  |  | -0.106 [-0.452; 0.240] | 0.545 |  |  |  |  | -0.104 [-0.450; 0.242] | 0.552 |
| LTPA = 2 |  |  |  | -0.045 [-0.362; 0.272] | 0.779 |  |  |  |  | -0.044 [-0.361; 0.272] | 0.783 |
| LTPA = 3 |  |  |  | -0.406 [-0.843; 0.032] | 0.069 |  |  |  |  | -0.414 [-0.854; 0.025] | 0.064 |
| Smoking = 1 |  |  |  | 0.138 [-0.181; 0.457] | 0.394 |  |  |  |  | 0.131 [-0.190; 0.452] | 0.421 |
| Smoking = 2 |  |  |  | -0.222 [-0.538; 0.094] | 0.167 |  |  |  |  | -0.226 [-0.543; 0.092] | 0.162 |
| Comorbidity = 1 |  |  |  |  |  |  | 0.306 [-0.083; 0.696] | 0.122 |  | 0.289 [-0.115; 0.694] | 0.160 |
| Medication = 1 |  |  |  |  |  |  | -0.188 [-0.510; 0.135] | 0.253 |  | -0.209 [-0.544; 0.126] | 0.220 |
|  |  |  |  |  |  |  |  |  |  |  |  |
| **Outcome: SBPV, standing** |  |  |  |  |  |  |  |  |  |  |  |
| NPS | 0.009 [-0.053; 0.070] | 0.784 |  | 0.018 [-0.045; 0.081] | 0.571 |  | 0.015 [-0.048; 0.079] | 0.632 |  | 0.024 [-0.041; 0.088] | 0.474 |
| BMI |  |  |  | -0.011 [-0.040; 0.018] | 0.458 |  |  |  |  | -0.009 [-0.039; 0.022] | 0.569 |
| LTPA = 1 |  |  |  | 0.150 [-0.199; 0.499] | 0.397 |  |  |  |  | 0.160 [-0.191; 0.511] | 0.368 |
| LTPA = 2 |  |  |  | 0.078 [-0.242; 0.398] | 0.631 |  |  |  |  | 0.071 [-0.250; 0.393] | 0.661 |
| LTPA = 3 |  |  |  | -0.444 [-0.885; -0.002] | 0.049 |  |  |  |  | -0.466 [-0.911; -0.020] | 0.041 |
| Smoking = 1 |  |  |  | 0.081 [-0.241; 0.403] | 0.621 |  |  |  |  | 0.089 [-0.237; 0.415] | 0.589 |
| Smoking = 2 |  |  |  | -0.280 [-0.599; 0.038] | 0.084 |  |  |  |  | -0.271 [-0.592; 0.051] | 0.099 |
| Comorbidity = 1 |  |  |  |  |  |  | 0.154 [-0.244; 0.553] | 0.446 |  | 0.124 [-0.287; 0.534] | 0.553 |
| Medication = 1 |  |  |  |  |  |  | -0.168 [-0.498; 0.163] | 0.318 |  | -0.211 [-0.551; 0.128] | 0.221 |
|  |  |  |  |  |  |  |  |  |  |  |  |
| **Outcome: BRS, seated** |  |  |  |  |  |  |  |  |  |  |  |
| NPS | 0.023 [-0.019; 0.065] | 0.282 |  | 0.042 [0.002; 0.082] | 0.039 |  | 0.012 [-0.030; 0.054] | 0.569 |  | 0.032 [-0.009; 0.072] | 0.130 |
| BMI |  |  |  | -0.016 [-0.034; 0.002] | 0.090 |  |  |  |  | -0.010 [-0.029; 0.009] | 0.311 |
| LTPA = 1 |  |  |  | 0.144 [-0.078; 0.366] | 0.203 |  |  |  |  | 0.159 [-0.062; 0.380] | 0.157 |
| LTPA = 2 |  |  |  | 0.174 [-0.030; 0.377] | 0.093 |  |  |  |  | 0.160 [-0.042; 0.363] | 0.119 |
| LTPA = 3 |  |  |  | 0.520 [0.239; 0.800] | < 0.001 |  |  |  |  | 0.494 [0.213; 0.774] | 0.001 |
| Smoking = 1 |  |  |  | -0.351 [-0.556; -0.146] | 0.001 |  |  |  |  | -0.324 [-0.529; -0.119] | 0.002 |
| Smoking = 2 |  |  |  | -0.210 [-0.413; -0.007] | 0.042 |  |  |  |  | -0.186 [-0.388; 0.017] | 0.072 |
| Comorbidity = 1 |  |  |  |  |  |  | -0.398 [-0.661; -0.134] | 0.003 |  | -0.276 [-0.535; -0.018] | 0.036 |
| Medication = 1 |  |  |  |  |  |  | -0.132 [-0.350; 0.086] | 0.234 |  | -0.026 [-0.240; 0.189] | 0.814 |
|  |  |  |  |  |  |  |  |  |  |  |  |
| **Outcome: BRS, standing** |  |  |  |  |  |  |  |  |  |  |  |
| NPS | 0.027 [-0.019; 0.072] | 0.252 |  | 0.047 [0.002; 0.092] | 0.039 |  | 0.016 [-0.030; 0.062] | 0.485 |  | 0.038 [-0.008; 0.084] | 0.108 |
| BMI |  |  |  | -0.021 [-0.042; -0.001] | 0.043 |  |  |  |  | -0.016 [-0.038; 0.006] | 0.143 |
| LTPA = 1 |  |  |  | 0.015 [-0.234; 0.264] | 0.906 |  |  |  |  | 0.028 [-0.222; 0.278] | 0.824 |
| LTPA = 2 |  |  |  | 0.150 [-0.078; 0.379] | 0.196 |  |  |  |  | 0.139 [-0.090; 0.367] | 0.232 |
| LTPA = 3 |  |  |  | 0.362 [0.047; 0.677] | 0.025 |  |  |  |  | 0.340 [0.023; 0.657] | 0.036 |
| Smoking = 1 |  |  |  | -0.323 [-0.553; -0.093] | 0.006 |  |  |  |  | -0.299 [-0.531; -0.067] | 0.012 |
| Smoking = 2 |  |  |  | -0.291 [-0.518; -0.063] | 0.013 |  |  |  |  | -0.270 [-0.499; -0.041] | 0.021 |
| Comorbidity = 1 |  |  |  |  |  |  | -0.380 [-0.669; -0.091] | 0.010 |  | -0.245 [-0.537; 0.047] | 0.099 |
| Medication = 1 |  |  |  |  |  |  | -0.129 [-0.369; 0.111] | 0.289 |  | -0.013 [-0.255; 0.229] | 0.917 |
