# Supplementary material for: Musculoskeletal pains and cardiovascular autonomic function in the general Northern Finnish population
Source: BMC Musculoskelet Disord. 2019 Jan 31;20:45. doi: 10.1186/s12891-019-2426-2 (PMC6357438; doi:10.1186/s12891-019-2426-2)
Supplement: Supplementary file 9 — Subanalysis 2, women. (DOCX 48. kb) [file 12891_2019_2426_MOESM9_ESM.docx]

**Additional file 9*.*** Subanalysis 2, women. Complete linear regression models for the association between number of pain sites (NPS) and cardiovascular autonomic function (HR, rMSSD, SBPV, BRS) among women in Subsample 2, i.e. women with clinically relevant symptoms of depression and anxiety (HSCL-25 score ≥ 1.55) (for HR and rMSSD, n = 507; for SBPV and BRS, n = 241). Variable coding, reference groups and model construction are presented in Additional files 1–3.

| Variables | Model I |  |  | Model II |  |  | Model III |  |  | Model IV |  |
| --- | --- | --- | --- | --- | --- | --- | --- | --- | --- | --- | --- |
|  | β [95% CI] | P |  | β [95% CI] | P |  | β [95% CI] | P |  | β [95% CI] | P |
| **Outcome: HR, seated** |  |  |  |  |  |  |  |  |  |  |  |
| NPS | 0.434 [-0.018; 0.885] | 0.060 |  | 0.265 [-0.163; 0.694] | 0.225 |  | 0.383 [-0.072; 0.837] | 0.099 |  | 0.267 [-0.164; 0.698] | 0.225 |
| BMI |  |  |  | 0.429 [0.257; 0.601] | < 0.001 |  |  |  |  | 0.431 [0.250; 0.611] | < 0.001 |
| LTPA = 1 |  |  |  | -2.553 [-5.059; -0.047] | 0.046 |  |  |  |  | -2.577 [-5.113; -0.041] | 0.046 |
| LTPA = 2 |  |  |  | -5.802 [-8.213; -3.392] | < 0.001 |  |  |  |  | -5.779 [-8.196; -3.362] | < 0.001 |
| LTPA = 3 |  |  |  | -4.754 [-7.985; -1.524] | 0.004 |  |  |  |  | -4.792 [-8.037; -1.547] | 0.004 |
| Smoking = 1 |  |  |  | -3.116 [-5.363; -0.869] | 0.007 |  |  |  |  | -3.099 [-5.355; -0.843] | 0.007 |
| Smoking = 2 |  |  |  | 0.025 [-2.296; 2.345] | 0.983 |  |  |  |  | 0.041 [-2.285; 2.367] | 0.972 |
| Comorbidity = 1 |  |  |  |  |  |  | 2.701 [-0.419; 5.821] | 0.090 |  | 0.385 [-2.649; 3.418] | 0.803 |
| Medication = 1 |  |  |  |  |  |  | 0.659 [-1.791; 3.108] | 0.597 |  | -0.446 [-2.825; 1.933] | 0.713 |
|  |  |  |  |  |  |  |  |  |  |  |  |
| **Outcome: HR, standing** |  |  |  |  |  |  |  |  |  |  |  |
| NPS | 0.586 [0.059; 1.112] | 0.029 |  | 0.422 [-0.086; 0.931] | 0.103 |  | 0.561 [0.030; 1.092] | 0.039 |  | 0.440 [-0.071; 0.952] | 0.091 |
| BMI |  |  |  | 0.382 [0.179; 0.586] | < 0.001 |  |  |  |  | 0.406 [0.192; 0.620] | < 0.001 |
| LTPA = 1 |  |  |  | -2.500 [-5.472; 0.472] | 0.099 |  |  |  |  | -2.657 [-5.663; 0.350] | 0.083 |
| LTPA = 2 |  |  |  | -6.436 [-9.294; -3.577] | < 0.001 |  |  |  |  | -6.412 [-9.278; -3.546] | < 0.001 |
| LTPA = 3 |  |  |  | -5.479 [-9.310; -1.647] | 0.005 |  |  |  |  | -5.567 [-9.414; -1.719] | 0.005 |
| Smoking = 1 |  |  |  | -3.852 [-6.517; -1.187] | 0.005 |  |  |  |  | -3.894 [-6.569; -1.220] | 0.004 |
| Smoking = 2 |  |  |  | -1.490 [-4.242; 1.262] | 0.288 |  |  |  |  | -1.483 [-4.240; 1.274] | 0.291 |
| Comorbidity = 1 |  |  |  |  |  |  | 1.421 [-2.225; 5.067] | 0.444 |  | -0.838 [-4.434; 2.758] | 0.647 |
| Medication = 1 |  |  |  |  |  |  | 0.215 [-2.647; 3.077] | 0.883 |  | -0.760 [-3.581; 2.061] | 0.597 |
|  |  |  |  |  |  |  |  |  |  |  |  |
| **Outcome: rMSSD, seated** |  |  |  |  |  |  |  |  |  |  |  |
| NPS | -0.016 [-0.042; 0.010] | 0.237 |  | -0.005 [-0.030; 0.020] | 0.687 |  | -0.009 [-0.036; 0.017] | 0.479 |  | -0.003 [-0.028; 0.022] | 0.815 |
| BMI |  |  |  | -0.032 [-0.042; -0.022] | < 0.001 |  |  |  |  | -0.029 [-0.040; -0.019] | < 0.001 |
| LTPA = 1 |  |  |  | 0.130 [-0.015; 0.275] | 0.079 |  |  |  |  | 0.111 [-0.035; 0.257] | 0.137 |
| LTPA = 2 |  |  |  | 0.241 [0.101; 0.380] | 0.001 |  |  |  |  | 0.244 [0.104; 0.384] | 0.001 |
| LTPA = 3 |  |  |  | 0.235 [0.048; 0.423] | 0.014 |  |  |  |  | 0.224 [0.037; 0.411] | 0.019 |
| Smoking = 1 |  |  |  | 0.098 [-0.032; 0.228] | 0.139 |  |  |  |  | 0.094 [-0.036; 0.224] | 0.158 |
| Smoking = 2 |  |  |  | -0.073 [-0.207; 0.062] | 0.289 |  |  |  |  | -0.071 [-0.206; 0.063] | 0.297 |
| Comorbidity = 1 |  |  |  |  |  |  | -0.227 [-0.406; -0.047] | 0.013 |  | -0.086 [-0.261; 0.089] | 0.336 |
| Medication = 1 |  |  |  |  |  |  | -0.181 [-0.322; -0.040] | 0.012 |  | -0.103 [-0.240; 0.035] | 0.143 |
|  |  |  |  |  |  |  |  |  |  |  |  |
| **Outcome: rMSSD, standing** |  |  |  |  |  |  |  |  |  |  |  |
| NPS | -0.018 [-0.044; 0.009] | 0.187 |  | -0.007 [-0.032; 0.018] | 0.600 |  | -0.010 [-0.036; 0.016] | 0.449 |  | -0.004 [-0.029; 0.021] | 0.782 |
| BMI |  |  |  | -0.031 [-0.041; -0.021] | < 0.001 |  |  |  |  | -0.027 [-0.038; -0.017] | < 0.001 |
| LTPA = 1 |  |  |  | 0.123 [-0.023; 0.270] | 0.098 |  |  |  |  | 0.097 [-0.050; 0.244] | 0.196 |
| LTPA = 2 |  |  |  | 0.230 [0.089; 0.370] | 0.001 |  |  |  |  | 0.231 [0.091; 0.371] | 0.001 |
| LTPA = 3 |  |  |  | 0.214 [0.026; 0.403] | 0.026 |  |  |  |  | 0.202 [0.014; 0.390] | 0.035 |
| Smoking = 1 |  |  |  | 0.139 [0.008; 0.270] | 0.038 |  |  |  |  | 0.129 [-0.002; 0.260] | 0.053 |
| Smoking = 2 |  |  |  | -0.068 [-0.203; 0.068] | 0.326 |  |  |  |  | -0.068 [-0.203; 0.066] | 0.320 |
| Comorbidity = 1 |  |  |  |  |  |  | -0.334 [-0.512; -0.155] | < 0.001 |  | -0.201 [-0.377; -0.025] | 0.025 |
| Medication = 1 |  |  |  |  |  |  | -0.162 [-0.302; -0.021] | 0.024 |  | -0.091 [-0.228; 0.047] | 0.197 |
|  |  |  |  |  |  |  |  |  |  |  |  |
| **Outcome: SBPV, seated** |  |  |  |  |  |  |  |  |  |  |  |
| NPS | 0.022 [-0.030; 0.073] | 0.408 |  | 0.030 [-0.022; 0.082] | 0.253 |  | 0.022 [-0.030; 0.075] | 0.402 |  | 0.027 [-0.026; 0.080] | 0.310 |
| BMI |  |  |  | -0.009 [-0.028; 0.011] | 0.373 |  |  |  |  | -0.008 [-0.029; 0.012] | 0.422 |
| LTPA = 1 |  |  |  | 0.238 [-0.064; 0.539] | 0.121 |  |  |  |  | 0.237 [-0.071; 0.545] | 0.130 |
| LTPA = 2 |  |  |  | 0.043 [-0.244; 0.330] | 0.769 |  |  |  |  | 0.034 [-0.254; 0.322] | 0.817 |
| LTPA = 3 |  |  |  | 0.315 [-0.051; 0.681] | 0.092 |  |  |  |  | 0.325 [-0.044; 0.693] | 0.084 |
| Smoking = 1 |  |  |  | 0.061 [-0.215; 0.337] | 0.665 |  |  |  |  | 0.056 [-0.221; 0.332] | 0.691 |
| Smoking = 2 |  |  |  | -0.126 [-0.396; 0.144] | 0.357 |  |  |  |  | -0.124 [-0.395; 0.146] | 0.365 |
| Comorbidity = 1 |  |  |  |  |  |  | -0.224 [-0.545; 0.098] | 0.171 |  | -0.146 [-0.481; 0.189] | 0.392 |
| Medication = 1 |  |  |  |  |  |  | 0.021 [-0.249; 0.291] | 0.880 |  | 0.105 [-0.178; 0.389] | 0.464 |
|  |  |  |  |  |  |  |  |  |  |  |  |
| **Outcome: SBPV, standing** |  |  |  |  |  |  |  |  |  |  |  |
| NPS | 0.034 [-0.015; 0.083] | 0.175 |  | 0.039 [-0.010; 0.088] | 0.121 |  | 0.034 [-0.016; 0.084] | 0.182 |  | 0.037 [-0.013; 0.088] | 0.147 |
| BMI |  |  |  | -0.002 [-0.021; 0.016] | 0.811 |  |  |  |  | -0.003 [-0.023; 0.017] | 0.765 |
| LTPA = 1 |  |  |  | 0.131 [-0.155; 0.418] | 0.368 |  |  |  |  | 0.139 [-0.155; 0.432] | 0.353 |
| LTPA = 2 |  |  |  | 0.067 [-0.206; 0.340] | 0.631 |  |  |  |  | 0.063 [-0.211; 0.338] | 0.649 |
| LTPA = 3 |  |  |  | 0.281 [-0.067; 0.629] | 0.114 |  |  |  |  | 0.288 [-0.064; 0.639] | 0.108 |
| Smoking = 1 |  |  |  | -0.159 [-0.421; 0.104] | 0.235 |  |  |  |  | -0.159 [-0.423; 0.104] | 0.235 |
| Smoking = 2 |  |  |  | -0.313 [-0.570; -0.056] | 0.017 |  |  |  |  | -0.313 [-0.571; -0.055] | 0.017 |
| Comorbidity = 1 |  |  |  |  |  |  | -0.059 [-0.367; 0.249] | 0.706 |  | -0.014 [-0.333; 0.305] | 0.930 |
| Medication = 1 |  |  |  |  |  |  | 0.005 [-0.253; 0.264] | 0.969 |  | 0.057 [-0.213; 0.327] | 0.676 |
|  |  |  |  |  |  |  |  |  |  |  |  |
| **Outcome: BRS, seated** |  |  |  |  |  |  |  |  |  |  |  |
| NPS | -0.040 [-0.070; -0.010] | 0.010 |  | -0.030 [-0.059; -0.001] | 0.044 |  | -0.029 [-0.059; 0.001] | 0.054 |  | -0.024 [-0.053; 0.005] | 0.104 |
| BMI |  |  |  | -0.024 [-0.035; -0.013] | < 0.001 |  |  |  |  | -0.018 [-0.029; -0.007] | 0.002 |
| LTPA = 1 |  |  |  | 0.105 [-0.064; 0.273] | 0.222 |  |  |  |  | 0.052 [-0.117; 0.222] | 0.543 |
| LTPA = 2 |  |  |  | 0.072 [-0.089; 0.233] | 0.378 |  |  |  |  | 0.075 [-0.083; 0.234] | 0.351 |
| LTPA = 3 |  |  |  | 0.066 [-0.139; 0.271] | 0.525 |  |  |  |  | 0.039 [-0.164; 0.242] | 0.703 |
| Smoking = 1 |  |  |  | 0.123 [-0.031; 0.277] | 0.117 |  |  |  |  | 0.117 [-0.035; 0.269] | 0.132 |
| Smoking = 2 |  |  |  | -0.103 [-0.254; 0.048] | 0.180 |  |  |  |  | -0.099 [-0.247; 0.050] | 0.192 |
| Comorbidity = 1 |  |  |  |  |  |  | -0.307 [-0.488; -0.126] | 0.001 |  | -0.213 [-0.397; -0.029] | 0.023 |
| Medication = 1 |  |  |  |  |  |  | -0.228 [-0.380; -0.076] | 0.003 |  | -0.170 [-0.325; -0.014] | 0.033 |
|  |  |  |  |  |  |  |  |  |  |  |  |
| **Outcome: BRS, standing** |  |  |  |  |  |  |  |  |  |  |  |
| NPS | -0.053 [-0.087; -0.019] | 0.002 |  | -0.037 [-0.068; -0.006] | 0.021 |  | -0.038 [-0.071; -0.005] | 0.025 |  | -0.028 [-0.059; 0.003] | 0.074 |
| BMI |  |  |  | -0.038 [-0.050; -0.026] | < 0.001 |  |  |  |  | -0.031 [-0.043; -0.019] | < 0.001 |
| LTPA = 1 |  |  |  | 0.049 [-0.131; 0.229] | 0.590 |  |  |  |  | -0.013 [-0.193; 0.167] | 0.887 |
| LTPA = 2 |  |  |  | 0.135 [-0.036; 0.307] | 0.121 |  |  |  |  | 0.144 [-0.024; 0.312] | 0.093 |
| LTPA = 3 |  |  |  | 0.219 [0.001; 0.438] | 0.049 |  |  |  |  | 0.182 [-0.033; 0.397] | 0.097 |
| Smoking = 1 |  |  |  | 0.220 [0.055; 0.384] | 0.009 |  |  |  |  | 0.214 [0.053; 0.376] | 0.009 |
| Smoking = 2 |  |  |  | 0.053 [-0.108; 0.214] | 0.514 |  |  |  |  | 0.058 [-0.100; 0.215] | 0.472 |
| Comorbidity = 1 |  |  |  |  |  |  | -0.314 [-0.516; -0.112] | 0.002 |  | -0.177 [-0.372; 0.018] | 0.075 |
| Medication = 1 |  |  |  |  |  |  | -0.350 [-0.519; -0.180] | < 0.001 |  | -0.258 [-0.423; -0.093] | 0.002 |
