# Supplementary material for: Musculoskeletal pains and cardiovascular autonomic function in the general Northern Finnish population
Source: BMC Musculoskelet Disord. 2019 Jan 31;20:45. doi: 10.1186/s12891-019-2426-2 (PMC6357438; doi:10.1186/s12891-019-2426-2)
Supplement: Supplementary file 10 — Subanalysis 3, men. (DOCX 52 kb) [file 12891_2019_2426_MOESM10_ESM.docx]

**Additional file 10.** Subanalysis 3, men. Complete linear regression models for the association between musculoskeletal pain intensity (according to NRS) and cardiovascular autonomic function (HR, rMSSD, SBPV, BRS) among men (for HR and rMSSD, n = 1619; for SBPV and BRS, n = 832). Variable coding, reference groups and model construction are presented in Additional files 1–3.

| Variables | Model I | | |  | Model II | | |  | Model III | | |  | Model IV | | |
| --- | --- | --- | --- | --- | --- | --- | --- | --- | --- | --- | --- | --- | --- | --- | --- |
|  | β [95% CI] |  | P |  | β [95% CI] |  | P |  | β [95% CI] |  | P |  | β [95% CI] |  | P |
| **Outcome: HR, seated** |  |  |  |  |  |  |  |  |  |  |  |  |  |  |  |
| NRS | 0.427 [0.200; 0.655] |  | < 0.001 |  | 0.201 [-0.018; 0.421] |  | 0.072 |  | 0.348 [0.118; 0.578] |  | 0.003 |  | 0.172 [-0.050; 0.395] |  | 0.129 |
| BMI |  |  |  |  | 0.574 [0.433; 0.716] |  | < 0.001 |  |  |  |  |  | 0.566 [0.419; 0.712] |  | < 0.001 |
| LTPA = 1 |  |  |  |  | -2.021 [-3.648; -0.395] |  | 0.015 |  |  |  |  |  | -1.888 [-3.520; -0.257] |  | 0.023 |
| LTPA = 2 |  |  |  |  | -4.134 [-5.573; -2.695] |  | < 0.001 |  |  |  |  |  | -4.020 [-5.465; -2.575] |  | < 0.001 |
| LTPA = 3 |  |  |  |  | -5.984 [-7.830; -4.137] |  | < 0.001 |  |  |  |  |  | -5.829 [-7.685; -3.974] |  | < 0.001 |
| Smoking = 1 |  |  |  |  | 0.471 [-0.843; 1.785] |  | 0.482 |  |  |  |  |  | 0.395 [-0.921; 1.711] |  | 0.556 |
| Smoking = 2 |  |  |  |  | 3.222 [1.719; 4.725] |  | < 0.001 |  |  |  |  |  | 2.972 [1.452; 4.491] |  | < 0.001 |
| HSCL-25 |  |  |  |  |  |  |  |  | 3.683 [1.688; 5.677] |  | < 0.001 |  | 1.890 [-0.059; 3.838] |  | 0.057 |
| Comorbidity = 1 |  |  |  |  |  |  |  |  | 3.155 [1.082; 5.228] |  | 0.003 |  | 0.816 [-1.220; 2.853] |  | 0.432 |
| Medication = 1 |  |  |  |  |  |  |  |  | 0.393 [-1.281; 2.067] |  | 0.645 |  | -0.742 [-2.368; 0.884] |  | 0.371 |
|  |  |  |  |  |  |  |  |  |  |  |  |  |  |  |  |
| **Outcome: HR, standing** |  |  |  |  |  |  |  |  |  |  |  |  |  |  |  |
| NRS | 0.460 [0.209; 0.710] | | < 0.001 |  | 0.277 [0.030; 0.525] |  | 0.028 |  | 0.375 [0.121; 0.629] |  | 0.004 |  | 0.237 [-0.014; 0.487] |  | 0.064 |
| BMI |  | |  |  | 0.337 [0.179; 0.496] |  | < 0.001 |  |  |  |  |  | 0.350 [0.185; 0.515] |  | < 0.001 |
| LTPA = 1 |  |  |  |  | -1.943 [-3.774; -0.111] |  | 0.038 |  |  |  |  |  | -1.722 [-3.557; 0.113] |  | 0.066 |
| LTPA = 2 |  |  |  |  | -4.251 [-5.872; -2.630] |  | < 0.001 |  |  |  |  |  | -4.135 [-5.760; -2.510] |  | < 0.001 |
| LTPA = 3 |  | |  |  | -6.276 [-8.356; -4.196] |  | < 0.001 |  |  |  |  |  | -6.121 [-8.207; -4.034] |  | < 0.001 |
| Smoking = 1 |  | |  |  | 0.542 [-0.938; 2.022] |  | 0.473 |  |  |  |  |  | 0.460 [-1.020; 1.940] |  | 0.542 |
| Smoking = 2 |  | |  |  | 3.248 [1.556; 4.941] |  | < 0.001 |  |  |  |  |  | 2.914 [1.205; 4.623] |  | 0.001 |
| HSCL-25 |  | |  |  |  |  |  |  | 4.306 [2.102; 6.511] |  | < 0.001 |  | 2.639 [0.447; 4.830] |  | 0.018 |
| Comorbidity = 1 |  | |  |  |  |  |  |  | 2.340 [0.049; 4.631] |  | 0.045 |  | 0.588 [-1.702; 2.877] |  | 0.615 |
| Medication = 1 |  | |  |  |  |  |  |  | -1.154 [-3.004; 0.696] |  | 0.221 |  | -1.980 [-3.808; -0.151] |  | 0.034 |
|  |  | |  |  |  |  |  |  |  |  |  |  |  |  |  |
| **Outcome: rMSSD, seated** |  | |  |  |  |  |  |  |  |  |  |  |  |  |  |
| NRS | -0.024 [-0.036; -0.012] | | < 0.001 |  | -0.012 [-0.023; -0.000] |  | 0.044 |  | -0.020 [-0.032; -0.008] |  | 0.001 |  | -0.011 [-0.023; 0.001] |  | 0.064 |
| BMI |  | |  |  | -0.033 [-0.040; -0.026] |  | < 0.001 |  |  |  |  |  | -0.032 [-0.040; -0.025] |  | < 0.001 |
| LTPA = 1 |  | |  |  | 0.102 [0.016; 0.188] |  | 0.019 |  |  |  |  |  | 0.100 [0.014; 0.186] |  | 0.022 |
| LTPA = 2 |  | |  |  | 0.174 [0.098; 0.249] |  | < 0.001 |  |  |  |  |  | 0.171 [0.094; 0.247] |  | < 0.001 |
| LTPA = 3 |  | |  |  | 0.276 [0.179; 0.373] |  | < 0.001 |  |  |  |  |  | 0.272 [0.174; 0.369] |  | < 0.001 |
| Smoking = 1 |  | |  |  | -0.021 [-0.090; 0.048] |  | 0.544 |  |  |  |  |  | -0.019 [-0.089; 0.050] |  | 0.583 |
| Smoking = 2 |  | |  |  | -0.150 [-0.229; -0.071] |  | < 0.001 |  |  |  |  |  | -0.147 [-0.227; -0.067] |  | < 0.001 |
| HSCL-25 |  | |  |  |  |  |  |  | -0.126 [-0.231; -0.021] |  | 0.019 |  | -0.037 [-0.140; 0.065] |  | 0.477 |
| Comorbidity = 1 |  | |  |  |  |  |  |  | -0.119 [-0.228; -0.010] |  | 0.032 |  | 0.006 [-0.101; 0.113] |  | 0.911 |
| Medication = 1 |  | |  |  |  |  |  |  | -0.089 [-0.176; -0.001] |  | 0.048 |  | -0.028 [-0.113; 0.058] |  | 0.526 |
|  |  | |  |  |  |  |  |  |  |  |  |  |  |  |  |
| **Outcome: rMSSD, standing** |  | |  |  |  |  |  |  |  |  |  |  |  |  |  |
| NRS | -0.020 [-0.032; -0.008] | | < 0.001 |  | -0.011 [-0.022; 0.001] |  | 0.075 |  | -0.016 [-0.028; -0.004] |  | 0.008 |  | -0.009 [-0.021; 0.002] |  | 0.118 |
| BMI |  | |  |  | -0.023 [-0.031; -0.016] |  | < 0.001 |  |  |  |  |  | -0.021 [-0.029; -0.014] |  | < 0.001 |
| LTPA = 1 |  | |  |  | 0.085 [-0.001; 0.171] |  | 0.054 |  |  |  |  |  | 0.082 [-0.004; 0.169] |  | 0.062 |
| LTPA = 2 |  | |  |  | 0.142 [0.066; 0.218] |  | < 0.001 |  |  |  |  |  | 0.136 [0.059; 0.212] |  | 0.001 |
| LTPA = 3 |  | |  |  | 0.237 [0.139; 0.334] |  | < 0.001 |  |  |  |  |  | 0.228 [0.129; 0.326] |  | < 0.001 |
| Smoking = 1 |  | |  |  | 0.004 [-0.065; 0.074] |  | 0.908 |  |  |  |  |  | 0.008 [-0.062; 0.078] |  | 0.819 |
| Smoking = 2 |  | |  |  | -0.171 [-0.251; -0.092] |  | < 0.001 |  |  |  |  |  | -0.163 [-0.244; -0.083] |  | < 0.001 |
| HSCL-25 |  | |  |  |  |  |  |  | -0.142 [-0.246; -0.038] |  | 0.007 |  | -0.065 [-0.168; 0.038] |  | 0.218 |
| Comorbidity = 1 |  | |  |  |  |  |  |  | -0.125 [-0.233; -0.017] |  | 0.023 |  | -0.033 [-0.141; 0.074] |  | 0.543 |
| Medication = 1 |  | |  |  |  |  |  |  | -0.086 [-0.173; 0.001] |  | 0.054 |  | -0.047 [-0.133; 0.039] |  | 0.284 |
|  |  | |  |  |  |  |  |  |  |  |  |  |  |  |  |
| **Outcome: SBPV, seated** |  | |  |  |  |  |  |  |  |  |  |  |  |  |  |
| NRS | 0.000 [-0.020; 0.021] | | 0.978 |  | 0.002 [-0.020; 0.023] |  | 0.882 |  | 0.004 [-0.017; 0.026] |  | 0.680 |  | 0.004 [-0.017; 0.026] |  | 0.691 |
| BMI |  | |  |  | 0.001 [-0.013; 0.015] |  | 0.878 |  |  |  |  |  | 0.001 [-0.013; 0.016] |  | 0.846 |
| LTPA = 1 |  | |  |  | -0.152 [-0.332; 0.027] |  | 0.096 |  |  |  |  |  | 0.027 [-0.132; 0.187] |  | 0.736 |
| LTPA = 2 |  | |  |  | 0.031 [-0.110; 0.172] |  | 0.668 |  |  |  |  |  | 0.024 [-0.118; 0.166] |  | 0.739 |
| LTPA = 3 |  | |  |  | 0.031 [-0.128; 0.189] |  | 0.702 |  |  |  |  |  | -0.159 [-0.340; 0.021] |  | 0.083 |
| Smoking = 1 |  | |  |  | -0.067 [-0.194; 0.061] |  | 0.307 |  |  |  |  |  | -0.060 [-0.198; 0.068] |  | 0.356 |
| Smoking = 2 |  | |  |  | -0.230 [-0.378; -0.082] |  | 0.002 |  |  |  |  |  | -0.219 [-0.369; -0.068] |  | 0.004 |
| HSCL-25 |  | |  |  |  |  |  |  | -0.139 [-0.326; 0.047] |  | 0.144 |  | -0.095 [-0.285; 0.095] |  | 0.326 |
| Comorbidity = 1 |  | |  |  |  |  |  |  | 0.064 [-0.143; 0.270] |  | 0.545 |  | 0.063 [-0.146; 0.273] |  | 0.554 |
| Medication = 1 |  | |  |  |  |  |  |  | -0.047 [-0.203; 0.108] |  | 0.551 |  | -0.057 [-0.214; 0.100] |  | 0.478 |
|  |  | |  |  |  |  |  |  |  |  |  |  |  |  |  |
| **Outcome: SBPV, standing** |  | |  |  |  |  |  |  |  |  |  |  |  |  |  |
| NRS | -0.006 [-0.027; 0.015] | | 0.596 |  | -0.004 [-0.025; 0.018] |  | 0.733 |  | 0.004 [-0.017; 0.026] |  | 0.687 |  | 0.002 [-0.019; 0.024] |  | 0.826 |
| BMI |  | |  |  | 0.005 [-0.009; 0.019] |  | 0.465 |  |  |  |  |  | 0.011 [-0.003; 0.025] |  | 0.136 |
| LTPA = 1 |  | |  |  | 0.112 [-0.046; 0.271] |  | 0.164 |  |  |  |  |  | 0.102 [-0.055; 0.260] |  | 0.203 |
| LTPA = 2 |  | |  |  | 0.084 [-0.058; 0.225] |  | 0.245 |  |  |  |  |  | 0.057 [-0.084; 0.197] |  | 0.429 |
| LTPA = 3 |  | |  |  | -0.054 [-0.234; 0.126] |  | 0.557 |  |  |  |  |  | -0.087 [-0.265; 0.092] |  | 0.341 |
| Smoking = 1 |  | |  |  | -0.057 [-0.185; 0.071] |  | 0.381 |  |  |  |  |  | -0.032 [-0.159; 0.096] |  | 0.627 |
| Smoking = 2 |  | |  |  | -0.268 [-0.416; -0.120] |  | < 0.001 |  |  |  |  |  | -0.222 [-0.371; -0.073] |  | 0.003 |
| HSCL-25 |  | |  |  |  |  |  |  | -0.347 [-0.532; -0.162] |  | < 0.001 |  | -0.295 [-0.483; -0.107] |  | 0.002 |
| Comorbidity = 1 |  | |  |  |  |  |  |  | -0.053 [-0.256; 0.150] |  | 0.608 |  | -0.074 [-0.280; 0.132] |  | 0.480 |
| Medication = 1 |  | |  |  |  |  |  |  | -0.195 [-0.349; -0.041] |  | 0.013 |  | -0.224 [-0.379; -0.068] |  | 0.005 |
|  |  | |  |  |  |  |  |  |  |  |  |  |  |  |  |
| **Outcome: BRS, seated** |  | |  |  |  |  |  |  |  |  |  |  |  |  |  |
| NRS | -0.019 [-0.032; -0.005] | | 0.008 |  | -0.006 [-0.019; 0.007] |  | 0.363 |  | -0.017 [-0.031; -0.003] |  | 0.015 |  | -0.007 [-0.020; 0.007] |  | 0.343 |
| BMI |  | |  |  | -0.036 [-0.045; -0.027] |  | < 0.001 |  |  |  |  |  | -0.034 [-0.044; -0.025] |  | < 0.001 |
| LTPA = 1 |  | |  |  | 0.067 [-0.032; 0.166] |  | 0.182 |  |  |  |  |  | 0.069 [-0.031; 0.168] |  | 0.175 |
| LTPA = 2 |  | |  |  | 0.090 [0.002; 0.178] |  | 0.045 |  |  |  |  |  | 0.087 [-0.002; 0.175] |  | 0.055 |
| LTPA = 3 |  | |  |  | 0.230 [0.117; 0.342] |  | < 0.001 |  |  |  |  |  | 0.225 [0.113; 0.338] |  | < 0.001 |
| Smoking = 1 |  | |  |  | -0.060 [-0.140; 0.020] |  | 0.143 |  |  |  |  |  | -0.057 [-0.137; 0.024] |  | 0.166 |
| Smoking = 2 |  | |  |  | -0.106 [-0.199; -0.014] |  | 0.025 |  |  |  |  |  | -0.102 [-0.196; -0.008] |  | 0.033 |
| HSCL-25 |  | |  |  |  |  |  |  | -0.065 [-0.186; 0.057] |  | 0.297 |  | -0.004 [-0.123; 0.114] |  | 0.943 |
| Comorbidity = 1 |  | |  |  |  |  |  |  | -0.172 [-0.307; -0.038] |  | 0.012 |  | -0.065 [-0.195; 0.066] |  | 0.333 |
| Medication = 1 |  | |  |  |  |  |  |  | -0.110 [-0.211; -0.009] |  | 0.033 |  | -0.047 [-0.145; 0.051] |  | 0.348 |
|  |  | |  |  |  |  |  |  |  |  |  |  |  |  |  |
| **Outcome: BRS, standing** |  | |  |  |  |  |  |  |  |  |  |  |  |  |  |
| NRS | -0.014 [-0.029; 0.000] | | 0.051 |  | -0.003 [-0.017; 0.011] |  | 0.682 |  | -0.013 [-0.027; 0.002] |  | 0.095 |  | -0.003 [-0.017; 0.012] |  | 0.710 |
| BMI |  | |  |  | -0.033 [-0.042; -0.023] |  | < 0.001 |  |  |  |  |  | -0.031 [-0.041; -0.022] |  | < 0.001 |
| LTPA = 1 |  | |  |  | 0.014 [-0.091; 0.119] |  | 0.794 |  |  |  |  |  | 0.012 [-0.094; 0.118] |  | 0.823 |
| LTPA = 2 |  | |  |  | 0.054 [-0.039; 0.148] |  | 0.254 |  |  |  |  |  | -0.051 [-0.044; 0.145] |  | 0.292 |
| LTPA = 3 |  | |  |  | 0.175 [0.056; 0.294] |  | 0.004 |  |  |  |  |  | 0.170 [0.050; 0.289] |  | 0.006 |
| Smoking = 1 |  | |  |  | -0.025 [-0.110; 0.060] |  | 0.568 |  |  |  |  |  | -0.021 [-0.106; 0.065] |  | 0.635 |
| Smoking = 2 |  | |  |  | -0.142 [-0.240; -0.044] |  | 0.005 |  |  |  |  |  | -0.134 [-0.234; -0.034] |  | 0.008 |
| HSCL-25 |  | |  |  |  |  |  |  | -0.093 [-0.221; 0.034] |  | 0.151 |  | -0.036 [-0.162; 0.090] |  | 0.579 |
| Comorbidity = 1 |  | |  |  |  |  |  |  | -0.164 [-0.305; -0.024] |  | 0.022 |  | -0.054 [-0.193; 0.085] |  | 0.447 |
| Medication = 1 |  | |  |  |  |  |  |  | -0.077 [-0.183; 0.030] |  | 0.157 |  | -0.026 [-0.131; 0.079] |  | 0.625 |
