# Supplementary material for: Musculoskeletal pains and cardiovascular autonomic function in the general Northern Finnish population
Source: BMC Musculoskelet Disord. 2019 Jan 31;20:45. doi: 10.1186/s12891-019-2426-2 (PMC6357438; doi:10.1186/s12891-019-2426-2)
Supplement: Supplementary file 11 — Subanalysis 3, women. (DOCX 52 kb) [file 12891_2019_2426_MOESM11_ESM.docx]

**Additional file 11.** Subanalysis 3, women. Complete linear regression models for the association between musculoskeletal pain intensity (according to NRS) and cardiovascular autonomic function (HR, rMSSD, SBPV, BRS) among women (for HR and rMSSD, n = 2096; for SBPV and BRS, n = 979). Variable coding, reference groups and model construction are presented in Additional files 1–3.

| Variables | Model I | | |  | Model II | | |  | Model III | | |  | Model IV | | |
| --- | --- | --- | --- | --- | --- | --- | --- | --- | --- | --- | --- | --- | --- | --- | --- |
|  | β [95% CI] |  | P |  | β [95% CI] |  | P |  | β [95% CI] |  | P |  | β [95% CI] |  | P |
| **Outcome: HR, seated** |  |  |  |  |  |  |  |  |  |  |  |  |  |  |  |
| NRS | 0.128 [-0.037; 0.294] |  | 0.129 |  | 0.048 [-0.115; 0.212] |  | 0.562 |  | 0.046 [-0.125; 0.218] |  | 0.597 |  | 0.010 [-0.159; 0.179] |  | 0.908 |
| BMI |  |  |  |  | 0.355 [0.264; 0.446] |  | < 0.001 |  |  |  |  |  | 0.331 [0.237; 0.426] |  | < 0.001 |
| LTPA = 1 |  |  |  |  | -0.430 [-1.738; 0.878] |  | 0.519 |  |  |  |  |  | -0.352 [-1.659; 0.955] |  | 0.597 |
| LTPA = 2 |  |  |  |  | -1.454 [-2.645; -0.263] |  | 0.017 |  |  |  |  |  | -1.354 [-2.547; -0.160] |  | 0.026 |
| LTPA = 3 |  |  |  |  | -3.970 [-5.410; -2.530] |  | < 0.001 |  |  |  |  |  | -3.875 [-5.320; -2.430] |  | < 0.001 |
| Smoking = 1 |  |  |  |  | -1.926 [-2.984; -0.868] |  | < 0.001 |  |  |  |  |  | -1.909 [-2.966; -0.851] |  | < 0.001 |
| Smoking = 2 |  |  |  |  | -0.640 [-1.872; 0.592] |  | 0.308 |  |  |  |  |  | -0.683 [-1.917; 0.551] |  | 0.278 |
| HSCL-25 |  |  |  |  |  |  |  |  | 0.816 [-0.605; 2.237] |  | 0.260 |  | 0.490 [-0.914; 1.895] |  | 0.494 |
| Comorbidity = 1 |  |  |  |  |  |  |  |  | 3.822 [2.220; 5.425] |  | < 0.001 |  | 2.699 [1.111; 4.287] |  | 0.001 |
| Medication = 1 |  |  |  |  |  |  |  |  | 0.898 [-0.397; 2.192] |  | 0.174 |  | -0.268 [-1.565; 1.029] |  | 0.685 |
|  |  |  |  |  |  |  |  |  |  |  |  |  |  |  |  |
| **Outcome: HR, standing** |  |  |  |  |  |  |  |  |  |  |  |  |  |  |  |
| NRS | 0.097 [-0.097; 0.290] | | 0.327 |  | 0.055 [-0.139; 0.248] |  | 0.581 |  | 0.049 [-0.153; 0.250] |  | 0.637 |  | 0.035 [-0.166; 0.236] |  | 0.733 |
| BMI |  | |  |  | 0.234 [0.126; 0.341] |  | < 0.001 |  |  |  |  |  | 0.227 [0.115; 0.339] |  | < 0.001 |
| LTPA = 1 |  |  |  |  | -0.399 [-1.949; 1.150] |  | 0.613 |  |  |  |  |  | -0.372 [-1.922; 1.179] |  | 0.638 |
| LTPA = 2 |  |  |  |  | -1.736 [-3.147; -0.325] |  | 0.016 |  |  |  |  |  | -1.658 [-3.074; -0.242] |  | 0.022 |
| LTPA = 3 |  | |  |  | -4.271 [-5.977; -2.656] |  | < 0.001 |  |  |  |  |  | -4.253 [-5.967; -2.539] |  | < 0.001 |
| Smoking = 1 |  | |  |  | -2.004 [-3.257; -0.751] |  | 0.002 |  |  |  |  |  | -1.987 [-3.324; -0.733] |  | 0.002 |
| Smoking = 2 |  | |  |  | -1.905 [-3.364; -0.446] |  | 0.011 |  |  |  |  |  | -1.905 [-3.368; -0.441] |  | 0.011 |
| HSCL-25 |  | |  |  |  |  |  |  | 0.471 [-1.198; 2.140] |  | 0.580 |  | 0.230 [-1.437; 1.896] |  | 0.787 |
| Comorbidity = 1 |  | |  |  |  |  |  |  | 2.886 [1.004; 4.768] |  | 0.003 |  | 2.059 [0.175; 3.942] |  | 0.032 |
| Medication = 1 |  | |  |  |  |  |  |  | -0.118 [-1.639; 1.402] |  | 0.879 |  | -0.952 [-2.490; 0.587] |  | 0.225 |
|  |  | |  |  |  |  |  |  |  |  |  |  |  |  |  |
| **Outcome: rMSSD, seated** |  | |  |  |  |  |  |  |  |  |  |  |  |  |  |
| NRS | -0.016 [-0.026; -0.006] | | 0.001 |  | -0.010 [-0.020; -0.001] |  | 0.036 |  | -0.009 [-0.018; 0.001] |  | 0.087 |  | -0.006 [-0.015; 0.004] |  | 0.257 |
| BMI |  | |  |  | -0.023 [-0.028; -0.018] |  | < 0.001 |  |  |  |  |  | -0.020 [-0.025; -0.014] |  | < 0.001 |
| LTPA = 1 |  | |  |  | 0.004 [-0.072; 0.079] |  | 0.927 |  |  |  |  |  | -0.006 [-0.081; 0.069] |  | 0.870 |
| LTPA = 2 |  | |  |  | 0.038 [-0.031; 0.106] |  | 0.285 |  |  |  |  |  | 0.029 [-0.039; 0.098] |  | 0.400 |
| LTPA = 3 |  | |  |  | 0.152 [0.069; 0.235] |  | < 0.001 |  |  |  |  |  | 0.136 [-0.053; 0.220] |  | 0.001 |
| Smoking = 1 |  | |  |  | 0.059 [-0.002; 0.120] |  | 0.059 |  |  |  |  |  | 0.059 [-0.002; 0.120] |  | 0.057 |
| Smoking = 2 |  | |  |  | -0.015 [-0.087; 0.056] |  | 0.670 |  |  |  |  |  | -0.007 [-0.078; 0.064] |  | 0.857 |
| HSCL-25 |  | |  |  |  |  |  |  | -0.089 [-0.170; -0.008] |  | 0.032 |  | -0.073 [-0.154; 0.008] |  | 0.076 |
| Comorbidity = 1 |  | |  |  |  |  |  |  | -0.242 [-0.334; -0.151] |  | < 0.001 |  | -0.180 [-0.272; -0.089] |  | < 0.001 |
| Medication = 1 |  | |  |  |  |  |  |  | -0.147 [-0.221; -0.073] |  | < 0.001 |  | -0.081 [-0.156; -0.006] |  | 0.033 |
|  |  | |  |  |  |  |  |  |  |  |  |  |  |  |  |
| **Outcome: rMSSD, standing** |  | |  |  |  |  |  |  |  |  |  |  |  |  |  |
| NRS | -0.014 [-0.024; -0.004] | | 0.004 |  | -0.010 [-0.019; 0.001] |  | 0.051 |  | -0.008 [-0.018; 0.002] |  | 0.103 |  | -0.006 [-0.016; 0.004] |  | 0.212 |
| BMI |  | |  |  | -0.016 [-0.021; -0.010] |  | < 0.001 |  |  |  |  |  | -0.013 [-0.019; -0.007] |  | < 0.001 |
| LTPA = 1 |  | |  |  | 0.001 [-0.077, 0.078] |  | 0.987 |  |  |  |  |  | -0.007 [-0.084; 0.070] |  | 0.857 |
| LTPA = 2 |  | |  |  | 0.050 [-0.020; 0.120] |  | 0.164 |  |  |  |  |  | 0.044 [-0.026; 0.115] |  | 0.219 |
| LTPA = 3 |  | |  |  | 0.154 [0.068; 0.239] |  | < 0.001 |  |  |  |  |  | 0.142 [0.057; 0.228] |  | 0.001 |
| Smoking = 1 |  | |  |  | 0.035 [-0.028; 0.097] |  | 0.275 |  |  |  |  |  | 0.034 [-0.028; 0.097] |  | 0.284 |
| Smoking = 2 |  | |  |  | -0.027 [-0.100; 0.046] |  | 0.463 |  |  |  |  |  | -0.021 [-0.094; 0.052] |  | 0.568 |
| HSCL-25 |  | |  |  |  |  |  |  | -0.064 [-0.147; 0.019] |  | 0.130 |  | -0.045 [-0.128; 0.038] |  | 0.290 |
| Comorbidity = 1 |  | |  |  |  |  |  |  | -0.203 [-0.296; -0.110] |  | < 0.001 |  | -0.160 [-0.254; -0.066] |  | 0.001 |
| Medication = 1 |  | |  |  |  |  |  |  | -0.106 [-0.182; -0.031] |  | 0.006 |  | -0.060 [-0.137; 0.016] |  | 0.123 |
|  |  | |  |  |  |  |  |  |  |  |  |  |  |  |  |
| **Outcome: SBPV, seated** |  | |  |  |  |  |  |  |  |  |  |  |  |  |  |
| NRS | -0.013 [-0.032; 0.006] | | 0.181 |  | -0.011 [-0.031; 0.008] |  | 0.240 |  | -0.010 [-0.029; 0.010] |  | 0.338 |  | -0.009 [-0.029; 0.010] |  | 0.315 |
| BMI |  | |  |  | 0.004 [-0.006; 0.015] |  | 0.410 |  |  |  |  |  | 0.006 [-0.005; 0.017] |  | 0.284 |
| LTPA = 1 |  | |  |  | 0.009 [-0.146; 0.164] |  | 0.907 |  |  |  |  |  | -0.001 [-0.157; 0.155] |  | 0.991 |
| LTPA = 2 |  | |  |  | 0.050 [-0.090; 0.190] |  | 0.485 |  |  |  |  |  | 0.046 [-0.096; 0.187] |  | 0.527 |
| LTPA = 3 |  | |  |  | 0.033 [-0.135; 0.201] |  | 0.700 |  |  |  |  |  | 0.022 [-0.147; 0.192] |  | 0.796 |
| Smoking = 1 |  | |  |  | -0.087 [-0.214; 0.039] |  | 0.176 |  |  |  |  |  | -0.085 [-0.211; 0.042] |  | 0.190 |
| Smoking = 2 |  | |  |  | -0.173 [-0.318; -0.028] |  | 0.019 |  |  |  |  |  | -0.169 [-0.315; -0.023] |  | 0.023 |
| HSCL-25 |  | |  |  |  |  |  |  | -0.059 [-0.226; 0.108] |  | 0.489 |  | -0.029 [-0.198; 0.141] |  | 0.740 |
| Comorbidity = 1 |  | |  |  |  |  |  |  | -0.018 [-0.189; 0.153] |  | 0.837 |  | -0.025 [-0.199; 0.149] |  | 0.775 |
| Medication = 1 |  | |  |  |  |  |  |  | -0.065 [-0.210; 0.081] |  | 0.386 |  | -0.080 [-0.299; 0.070] |  | 0.297 |
|  |  | |  |  |  |  |  |  |  |  |  |  |  |  |  |
| **Outcome: SBPV, standing** |  | |  |  |  |  |  |  |  |  |  |  |  |  |  |
| NRS | -0.002 [-0.021; 0.017] | | 0.846 |  | -0.001 [-0.021; 0.018] |  | 0.890 |  | 0.001 [-0.019; 0.021] |  | 0.908 |  | 0.001 [-0.019; 0.021] |  | 0.955 |
| BMI |  | |  |  | 0.014 [0.003; 0.024] |  | 0.011 |  |  |  |  |  | 0.015 [0.004; 0.026] |  | 0.008 |
| LTPA = 1 |  | |  |  | 0.046 [-0.110; 0.202] |  | 0.565 |  |  |  |  |  | 0.038 [-0.120; 0.195] |  | 0.639 |
| LTPA = 2 |  | |  |  | 0.137 [-0.004; 0.279] |  | 0.057 |  |  |  |  |  | 0.134 [-0.008; 0.277] |  | 0.065 |
| LTPA = 3 |  | |  |  | 0.045 [-0.125; 0.216] |  | 0.600 |  |  |  |  |  | 0.037 [-0.135; 0.209] |  | 0.676 |
| Smoking = 1 |  | |  |  | -0.171 [-0.299; -0.044] |  | 0.009 |  |  |  |  |  | -0.168 [-0.297; -0.040] |  | 0.010 |
| Smoking = 2 |  | |  |  | -0.247 [-0.393; -0.101] |  | 0.001 |  |  |  |  |  | -0.242 [-0.389; -0.096] |  | 0.001 |
| HSCL-25 |  | |  |  |  |  |  |  | -0.084 [-0.254; 0.085] |  | 0.330 |  | -0.035 [-0.206; 0.136] |  | 0.655 |
| Comorbidity = 1 |  | |  |  |  |  |  |  | 0.034 [-0.140; 0.208] |  | 0.701 |  | 0.011 [-0.165; 0.186] |  | 0.904 |
| Medication = 1 |  | |  |  |  |  |  |  | -0.043 [-0.192; 0.106] |  | 0.571 |  | -0.083 [-0.234; 0.068] |  | 0.281 |
|  |  | |  |  |  |  |  |  |  |  |  |  |  |  |  |
| **Outcome: BRS, seated** |  | |  |  |  |  |  |  |  |  |  |  |  |  |  |
| NRS | -0.006 [-0.017; 0.006] | | 0.315 |  | 0.000 [-0.011; 0.011] |  | 0.956 |  | 0.001 [-0.010; 0.013] |  | 0.825 |  | 0.003 [-0.008; 0.015] |  | 0.557 |
| BMI |  | |  |  | -0.020 [-0.025; -0.014] |  | < 0.001 |  |  |  |  |  | -0.017 [-0.023; -0.010] |  | < 0.001 |
| LTPA = 1 |  | |  |  | 0.032 [-0.059; 0.122] |  | 0.492 |  |  |  |  |  | 0.010 [-0.080; 0.101] |  | 0.823 |
| LTPA = 2 |  | |  |  | 0.013 [-0.069; 0.095] |  | 0.751 |  |  |  |  |  | 0.003 [-0.080; 0.085] |  | 0.951 |
| LTPA = 3 |  | |  |  | 0.105 [0.006; 0.203] |  | 0.037 |  |  |  |  |  | 0.082 [-0.016; 0.181] |  | 0.102 |
| Smoking = 1 |  | |  |  | 0.078 [0.005; 0.152] |  | 0.038 |  |  |  |  |  | 0.081 [0.008; 0.155] |  | 0.030 |
| Smoking = 2 |  | |  |  | -0.086 [-0.171; -0.001] |  | 0.046 |  |  |  |  |  | -0.078 [-0.163; 0.006] |  | 0.069 |
| HSCL-25 |  | |  |  |  |  |  |  | -0.061 [-0.159; 0.038] |  | 0.227 |  | -0.040 [-0.138; 0.059] |  | 0.428 |
| Comorbidity = 1 |  | |  |  |  |  |  |  | -0.173 [-0.274; -0.071] |  | 0.001 |  | -0.117 [-0.218; -0.016] |  | 0.024 |
| Medication = 1 |  | |  |  |  |  |  |  | -0.184 [-0.271; -0.098] |  | < 0.001 |  | -0.134 [-0.221; -0.047] |  | 0.003 |
|  |  | |  |  |  |  |  |  |  |  |  |  |  |  |  |
| **Outcome: BRS, standing** |  | |  |  |  |  |  |  |  |  |  |  |  |  |  |
| NRS | -0.016 [-0.028; -0.004] | | 0.012 |  | -0.009 [-0.021; 0.003] |  | 0.134 |  | -0.009 [-0.021; 0.004] |  | 0.186 |  | -0.006 [-0.018; 0.006] |  | 0.351 |
| BMI |  | |  |  | -0.027 [-0.033; -0.020] |  | < 0.001 |  |  |  |  |  | -0.024 [-0.031; -0.017] |  | < 0.001 |
| LTPA = 1 |  | |  |  | 0.003 [-0.094; 0.100] |  | 0.949 |  |  |  |  |  | -0.014 [-0.111; 0.083] |  | 0.775 |
| LTPA = 2 |  | |  |  | 0.027 [-0.061; 0.114] |  | 0.552 |  |  |  |  |  | 0.022 [-0.066; 0.110] |  | 0.620 |
| LTPA = 3 |  | |  |  | 0.199 [0.093; 0.304] |  | < 0.001 |  |  |  |  |  | 0.182 [0.076; 0.287] |  | 0.001 |
| Smoking = 1 |  | |  |  | 0.111 [0.032; 0.190] |  | 0.006 |  |  |  |  |  | 0.115 [0.036; 0.194] |  | 0.004 |
| Smoking = 2 |  | |  |  | -0.002 [-0.093; 0.088] |  | 0.957 |  |  |  |  |  | 0.004 [-0.087; 0.094] |  | 0.936 |
| HSCL-25 |  | |  |  |  |  |  |  | -0.062 [-0.170; 0.046] |  | 0.262 |  | -0.035 [-0.141; 0.070] |  | 0.511 |
| Comorbidity = 1 |  | |  |  |  |  |  |  | -0.094 [-0.204; 0.016] |  | 0.095 |  | -0.016 [-0.124; 0.092] |  | 0.773 |
| Medication = 1 |  | |  |  |  |  |  |  | -0.245 [-0.339; -0.151] |  | < 0.001 |  | -0.173 [-0.266; -0.080] |  | < 0.001 |
